# Prevalence and burden of multiple sclerosis-related fatigue: a systematic literature review

**DOI:** 10.1186/s12883-021-02396-1

**Published:** 2021-12-02

**Authors:** Abril Oliva Ramirez, Alexander Keenan, Olivia Kalau, Evelyn Worthington, Lucas Cohen, Sumeet Singh

**Affiliations:** 1 EVERSANA, Burlington, Ontario Canada; 2 Health Economics and Market Access, Janssen Research & Development, LLC, Titusville, NJ USA

**Keywords:** Multiple sclerosis, Fatigue, Burden of illness, Systematic review, Prevalence, Economic, Quality of life

## Abstract

**Background:**

Multiple sclerosis (MS) is a chronic, demyelinating disease of the central nervous system that results in progressive and irreversible disability. Fatigue is one of the most common MS-related symptoms and is characterized by a persistent lack of energy that impairs daily functioning. The burden of MS-related fatigue is complex and multidimensional, and to our knowledge, no systematic literature review has been conducted on this subject. The purpose of this study was to conduct a systematic literature review on the epidemiology and burden of fatigue in people with multiple sclerosis (pwMS).

**Methods:**

Systematic searches were conducted in MEDLINE, Embase, and Evidence-Based Medicine Reviews to identify relevant studies of fatigue in pwMS. English-language records published from 2010 to January 2020 that met predefined eligibility criteria were included. We initially selected studies that reported quality of life (QoL) and economic outcomes according to categories of fatigue (e.g., fatigued vs non-fatigued). Studies assessing associations between economic outcomes and fatigue as a continuous measure were later included to supplement the available data.

**Results:**

The search identified 8147 unique records, 54 of which met the inclusion criteria. Of these, 39 reported epidemiological outcomes, 11 reported QoL, and 9 reported economic outcomes. The supplementary screen for economic studies with fatigue as a continuous measure included an additional 20 records.

Fatigue prevalence in pwMS ranged from 36.5 to 78.0%. MS-related fatigue was consistently associated with significantly lower QoL. Results on the economic impact of fatigue were heterogeneous, but most studies reported a significant association between presence or severity of fatigue and employment status, capacity to work, and sick leave. There was a gap in evidence regarding the direct costs of MS-related fatigue and the burden experienced by caregivers of pwMS.

**Conclusion:**

Fatigue is a prevalent symptom in pwMS and is associated with considerable QoL and economic burden. There are gaps in the evidence related to the direct costs of MS-related fatigue and the burden of fatigue on caregivers. Addressing fatigue over the clinical course of the disease may improve health and economic outcomes for patients with MS.

**Supplementary Information:**

The online version contains supplementary material available at 10.1186/s12883-021-02396-1.

## Introduction

Multiple sclerosis (MS) is a chronic inflammatory disease that results in progressive demyelination in the central nervous system. Approximately 2.3 million people worldwide have been diagnosed with MS, with the highest prevalence in North America, Western Europe, and Australasia [1, 2]. The onset of MS usually occurs in early adulthood [2, 3], however, 3–5% of people with MS (pwMS) are diagnosed before this age [2, 4]. The clinical course of MS can be differentiated by disease history, progression of irreversible disability, and the presence or absence of acute disease relapses. Four courses of MS have been identified: clinically isolated syndrome (CIS), relapsing-remitting MS (RRMS), secondary progressive MS (SPMS), and primary progressive MS (PPMS). Approximately 85% of pwMS are initially diagnosed with RRMS, which may eventually progress to SPMS with or without superimposed relapses [5].

Fatigue, which can be defined as a “significant lack of physical and/or mental energy that is perceived by the individual or caretaker to interfere with usual or desired activity,” [6] is one of the most common and debilitating symptoms of MS. [2, 7–10] Several studies have been published describing the prevalence and impact of fatigue on QoL and employment [11–13]; however, to our knowledge, no systematic literature review (SLR) synthesizing the available evidence has been published. Therefore, the primary objective of the current report was to conduct an SLR synthesizing the published data on the prevalence, economic cost, and QoL burden of fatigue in pwMS.

## Methods

An SLR was conducted to identify primary studies reporting epidemiological, economic, or QoL-related outcomes of MS-related fatigue in patients with CIS, RRMS, and/or SPMS, based on a predefined search strategy. The SLR adhered to the methodological and reporting guidelines outlined in the Preferred Reporting Items for Systematic Reviews and Meta-Analysis (PRISMA) checklist [14].

### Search strategy

The search strategy was developed and performed by an experienced medical information specialist through an iterative process in consultation with the review team. Peer review was completed by a second information specialist using the Peer Review of Electronic Search Strategies (PRESS) Checklist [15]. Databases searched included Ovid MEDLINE®, including Epub Ahead of Print and In-Process & Other Non-Indexed Citations, Embase, and the following Evidence-Based Medicine Reviews (EBMR) databases: Health Technology Assessment, and the National Health Service (NHS) Economic Evaluation Database. All searches were performed on January 27, 2020.

The search incorporated controlled vocabulary (e.g., “Multiple Sclerosis”, “Incidence”, “Prevalence”, “Fatigue”) and keywords (e.g., “RRMS”, “occurrence”, “epidemiology”, “tired”, “cost”). Results were limited to the publication years 2000 to present and excluded conference abstracts published prior to 2018.

A comprehensive search of the grey literature was conducted using the Grey Matters checklist [16]. The following conference websites were also searched for relevant abstracts published within the past 2 years: American Academy of Neurology (AAN), American Neurology Association (ANA), Academy of Managed Care Pharmacy (AMCP), Americas Committee for Treatment and Research in Multiple Sclerosis (ACTRIMS), European Academy of Neurology (EAN), European Committee for Treatment and Research in Multiple Sclerosis (ECTRIMS), International Society for Pharmacoeconomics and Outcomes (ISPOR) America and Europe.

The reference lists of included articles were also reviewed, and records identified as potentially relevant were screened.

For additional details, please see Additional file 1.

### Study eligibility criteria

The predefined inclusion and exclusion criteria pertaining to the population, intervention, comparator, outcome, and study design (PICOS) are presented in Table 1. Studies were included that evaluated at least 70% patients with RRMS, SPMS, or CIS, and that reported at least one outcome related to the epidemiological burden, humanistic burden, and/or economic burden of MS-related fatigue. Eligibility criteria were initially designed to select for studies reporting fatigue as a categorical measure (i.e., fatigued vs. non-fatigued patients, or low vs. high levels of fatigue) and its relationship with relevant outcomes. However, the eligibility criteria were revised to include studies reporting economic outcomes that evaluated fatigue as a continuous measure due to the sparse data available from the categorical studies in this area.

**Table 1:** PICOS criteria for inclusion and exclusion of studies

Inclusion Criteria	Exclusion Criteria
***Population***	
• People with MS and fatigue	• Studies in which greater than 30% of subjects have MS types other than RRMS, SPMS, or CIS (e.g. PPMS, RIS) • Studies reporting fatigue as a continuous measure ^a^
***Intervention***	
• Any or none	• N/A
***Comparator***	
• Any or none	• N/A
***Outcomes***	
• Epidemiologic measures of MS-related fatigue (i.e., prevalence or incidence, current or projected) • Health resource utilization and costs (e.g., hospitalization, physician visits, drugs, assistive devices, long-term care) associated with MS-related fatigue • Lost productivity/income experienced by patients, caregivers, family members, society associated with MS-related fatigue • Community costs (e.g., personal support professionals, home care) associated with MS-related fatigue • Other costs (e.g., disability payments or other income support) associated with MS-related fatigue • Measures of patient-reported health-related quality of life (HRQoL) using a validated general health measure or disease-specific instrument	• Studies that do not report methodology for assessing or identifying fatigue • Studies that do not report an outcome of interest in relation to MS-related fatigue, e.g., ° Only overall health costs for MS reported ° Only isolated dimensions of HRQoL or patient function (e.g. gait, cognitive impairment, anxiety/depression) reported
***Study Design***	
• Primary studies (e.g., surveys, epidemiological studies, natural history and disease progression studies, observational studies, registries or other real-world studies, BOI studies, clinical trials, economic evaluations) reporting one or more of the above outcomes	• Opinions, editorials, narrative reviews
***Language***	
• Articles in English ^b^	• All non-English articles
***Publication types and time frame***	
• 2010-present • All publication types (peer-reviewed articles, grey literature such as reports from government or other organizations, conference abstracts) • Conference abstracts from the past 2 years only	• None

^a^Initially, only studies reporting fatigue as a categorical measure (i.e., fatigued vs. non-fatigued patients, or levels of fatigue) were included. However, the eligibility criteria were later revised to include studies that evaluated fatigue as a continuous measure for outcomes related to economic burden, due to the sparse data identified in this area from categorical studies

^b^Search was not restricted to English language studies, but non-English studies were excluded in study selection phase

*Abbreviations*: *BOI* burden of illness, *CIS* clinically isolated syndrome, *FACIT* Functional Assessment of Chronic Illness Therapy, *FSS* Fatigue Severity Scale, *HRQoL* health-related quality of life, *MFIS* Modified Fatigue Impact Scale, *MS* multiple sclerosis, *N/A* not applicable, *PPMS* primary progressive multiple sclerosis, *RCT* randomized controlled trial, *RIS* radiologically isolated syndrome, *RRMS* relapsing-remitting multiple sclerosis, *SLR* systematic literature review, *SPMS* secondary progressive multiple sclerosis, *VAS* visual analogue scale

### Study selection

Study screening was performed using the systematic review software DistillerSR (Evidence Partners, Ontario, Canada). Screening was conducted by two reviewers who independently reviewed the citation titles and abstracts identified in the literature search to assess study eligibility based on the predefined PICOS criteria. Potentially relevant records were then screened by two reviewers in full-text form. Reasons for exclusion were documented at the full-text stage and are provided in Additional file 2. Any disagreements during study screening were resolved by discussion or by a third independent reviewer.

### Data extraction

Details for selected articles were collected using a standardized data extraction template in Microsoft Excel. Data extraction was performed by a single reviewer and validated by a second reviewer. General study information (reference identification, first author last name, publication year, and country/region of study) was extracted, in addition to a predefined list of epidemiological, economic, and QoL outcomes.

### Data synthesis

When multiple publications reporting data from the same study were identified, the most comprehensive data were used. When multiple analyses were conducted in a single study, the analysis with the most robust design was selected to be included in the synthesis, based on the following hierarchy: multivariate regression analyses; univariate regression analyses; correlation analyses; and statistical tests of association (e.g., t-test, χ^2^ test).

## Results

### Identification and description of studies

**Fig. 1:**
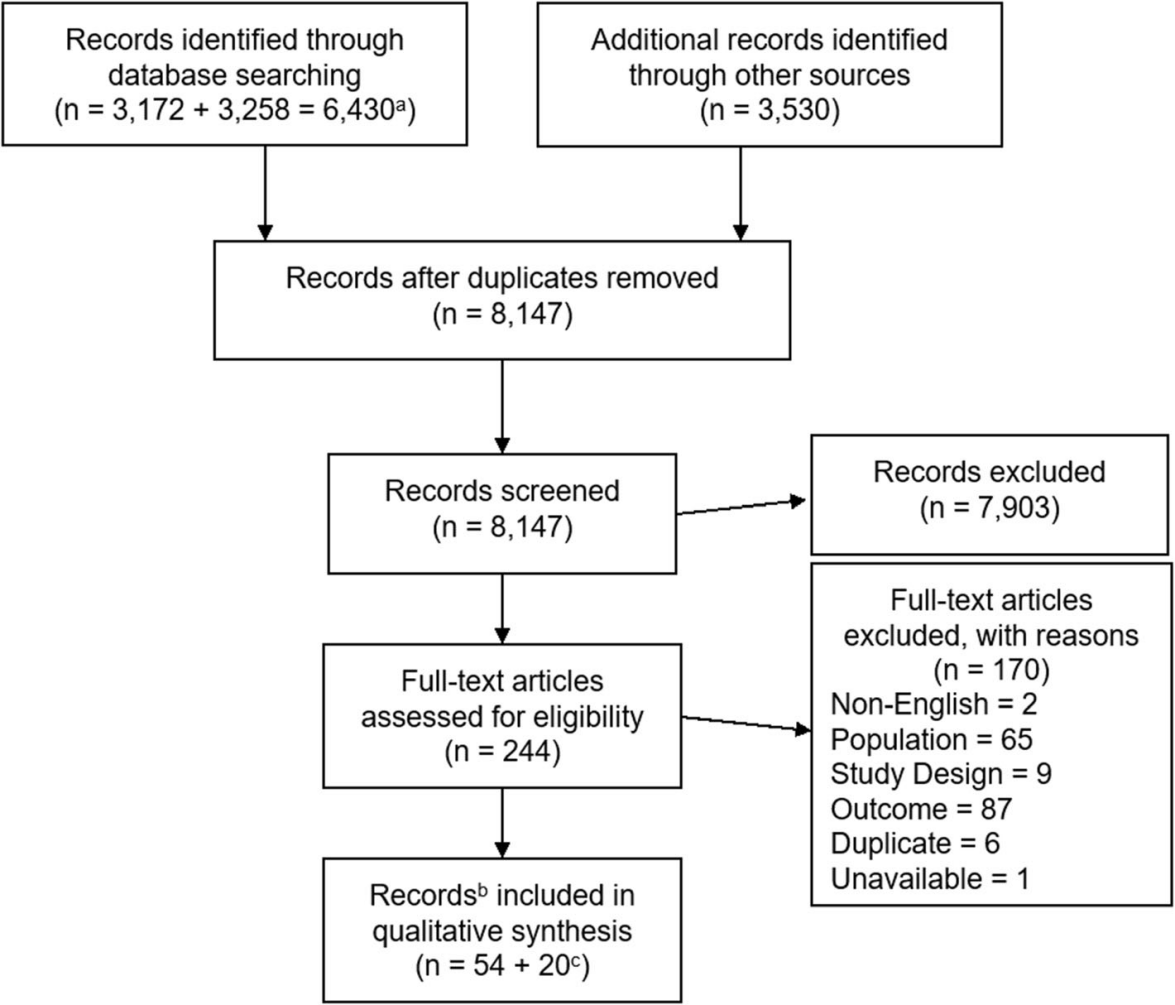
Search and exclusion process. ^a^ Searches were run separately for (1) epidemiology (*n* = 3172) and (2) economic/QoL studies (*n* = 3258). Each search was then deduplicated (epidemiology = 3081; economic/QoL = 3229). The two searches were then combined and deduplicated once again (*n* = 4631). ^**b**^ In some cases, more than one record was identified for a given study/population. ^c^ Supplemental search of economic studies with fatigue measured as a continuous parameter. Abbreviations: MA = meta-analysis; NMA = network meta-analysis; QoL = quality of life; SLR = systematic literature review

A total of 9960 records were identified through the database and grey literature searches. After de-duplication, 8147 records remained for title and abstract review. At the title and abstract stage, 244 full-text records were selected to be reviewed. Of these, 54 were found to fulfill the inclusion criteria. Results for each stage of the screening process are presented in Fig. 1. Of the included records, 40 (35 unique studies) examined epidemiological parameters (prevalence or incidence), nine investigated effects of fatigue on economic outcomes (costs, employment, etc.), and 11 investigated the effects of fatigue on QoL. Among these, one study reported data related to all three outcomes [11], two reported both epidemiology and QoL data [17, 18] and one reported both epidemiology and economic data [19]. An additional 20 records were identified through the supplementary screen for economic studies with fatigue as a continuous measure.

### Epidemiology

#### Prevalence

The SLR search identified 39 publications reporting the prevalence of fatigue in pwMS, based on 35 unique datasets (Additional file 3). Twenty-five studies used self-reported measurements administered in different settings (online, at the clinic, etc.) [11, 18, 20–42]. Physicians and/or researchers administered the assessment in four studies [17, 19, 43, 44] and seven studies did not specify an administration method [45–51]. Most studies (*n* = 25 studies) were conducted in Europe, North America, and Australasia [18, 21–24, 26–33, 36–39, 42, 43, 45, 48–52] and an additional five records were linked to the same international study in which most of the participants reported living in North America, Australasia, and Europe [11, 25, 34, 35, 40]. The remaining five datasets were from South America, the Middle East, or the region was not reported [17, 19, 20, 46, 47]. Sample sizes ranged from 26 to 5475 participants.

##### Adult population

Twenty-seven studies reported results for adults with MS. Across these studies, the prevalence of fatigue ranged from 18.2 to 97.0% (Table 2). The wide range of values reported was likely due to the considerable heterogeneity across studies in the instruments or criteria used to classify patients as having fatigue.

**Table 2:** Results – Epidemiology

Author (year)	Tool to Measure Fatigue; Cut-off Value Used	Outcome(s)	n evaluated for fatigue	Fatigue (%)
**Adult**				
Alvarenga-Filho (2015)	MFIS; ≥ 38	Prevalence	NR	35.0
Anens (2014)	FSS; ≥ 4	Prevalence	285	61.7
Battaglia (2017)	VAS (0–10); NR	Prevalence	997	96.0
Calabrese (2017)	VAS (0–10); NR	Prevalence	703	93.0
Fiest (2016)	D-FIS; ≥ 5.0	Prevalence, Incidence	943	78.0
Flachenecker (2017)	VAS (0–10); NR	Prevalence	5233	96.0
Fricska-Nagy (2016)	FIS; NR	Prevalence	402	62.4
Hadgkiss (2013)	FSS; ≥ 4	Prevalence	2143	65.7
Havrdova (2017)	VAS (0–10); NR	Prevalence	727	92.0
Kratz (2016)	11-point scale; Occurrence: > 0, Severe: > 6	Prevalence	180	88.0
Labuz-Roszak (2012)	FSS; > 36 ^a^	Prevalence	122	61.5
Larnaout (2018) ^b^	FSS; > 4, MFIS; > 38 ^c^	Prevalence	NR	60.0
Lebrun-Frenay (2017)	VAS (0–10); NR	Prevalence	454	95.0
Oreja-Guevara (2017)	VAS (0–10); NR	Prevalence	446	92.0
Pentek (2017)	VAS (0–10); NR	Prevalence	508	94.0
Pokryszko-Dragan (2016)	FSS; ≥ 4	Prevalence	44	18.2
Reilly (2017)	FSS; ≥ 4	Prevalence	2079	65.6
Rooney (2019)	FSS; ≥ 5	Prevalence	412	68.7
Runia (2015)	FSS; ≥ 5	Prevalence	127	46.5
Selmaj (2017)	VAS (0–10); NR	Prevalence	408	97.0
Thompson (2017)	VAS (0–10); NR	Prevalence	769	96.0
Uitdehaag (2017)	VAS (0–10); NR	Prevalence	381	96.0
van der Vuurst de Vries (2017)	FSS; ≥ 5	Prevalence	NR	35.3
von Bismarck (2018)	FSMC; At least mild fatigue (> 42 Pt.)	Prevalence	1069	36.5
Weiland (2015)	FSS; ≥4	Prevalence	2138	65.6
Weiland (2019) ^d^	FSS; ≥ 4	Prevalence, Longitudinal	**1268** 1268 *509*	**56.0** 62.5 *53.8*
Wood (2013)	FSS; ≥ 5	Prevalence	192	53.7
**Pediatric**				
Florea (2019)	FSS; Moderate ≥3	Prevalence	23	43.0
Goretti (2010)	FSS; ≥ 4	Prevalence	56	20.0
Parrish (2013)	PedsQL Multidimensional Fatigue Scale; Total Fatigue ≥36	Prevalence	24	29.2
van’s Gravesande (2019) ^b^	PedsQL Multidimensional Fatigue Scale; Mildly impaired: score 1–2 SDs below healthy controls, severely impaired: score > 2 SDs below healthy controls	Prevalence	106	40.6
**Mixed or unknown age**				
Garcia (2019) ^a, b, e^	FSS; Persistent fatigue ≥28, NFI-MS/BR; persistent fatigue ≥30	Prevalence, Longitudinal; Mixed age	**38** 26	FSS: **74.4,** 54.0 NFI-MS/BR: **64.0,** 47.0
Kaya Aygunoglu (2015)	FSS; ≥4	Prevalence	120	70.0
Razazian (2014)	FSS; ≥ 5	Prevalence	300	62.3
Rupprecht (2018) ^b^	MFIS; NR	Prevalence	NR	45.0

^a^Refers to the total FSS score, not the average as is mostly calculated

^b^Conference abstract

^c^Unclear if FSS or MFIS was used to report fatigue percentage

^d^Baseline data are presented in **bold** text and validation cohort in *italics*

^e^Baseline data are presented in **bold** text

*Abbreviations*: *D-FIS* daily FIS, *FIS* Fatigue Impact Scale, *FSS* Fatigue Severity Scale, *MFIS* modified FIS, *FSMC* Fatigue Scale for Motor and Cognitive Functions, *NFIS-MS/BR* Neurological Fatigue Index – multiple sclerosis, Brazilian Portuguese version, *NR* not reported, *VAS* visual analogue scale

##### *Prevalence of Fatigue Reported by Different Scales:*

Eleven studies did not use fatigue-specific validated instruments in estimating prevalence; ten studies used a 10-point visual analogue scale (VAS, cut-off not reported) and one study used an 11-point scale (fatigue presence defined as a score over 0) to measure fatigue. The prevalence of fatigue in these 11 studies ranged from 88.0 to 97.0% (Table 2). Validated fatigue scales (Table 3) were used in 16 studies (17 datasets) to define fatigue: seven studies used the Fatigue Severity Scale (FSS) ≥ 4, four used FSS ≥ 5, one used FSS >  36, two used the Modified Fatigue Impact Scale (MFIS) ≥ 38, one used the daily Fatigue Impact Scale (D-FIS) ≥ 5, one used the Fatigue Scale for Motor and Cognitive Functions (FSMC) >  42, and one used the Fatigue Impact Scale (FIS) with no cut-off reported. In these studies, the prevalence of fatigue ranged from 18.2 to 78.0%.

**Table 3:** Characteristics of validated fatigue scales

Validated fatigue scales	Domains/Components	Range of possible scores	Cut-offs for defining clinically relevant fatigue
Fatigue Severity Scale (FSS)	9 items: activities of daily living, life participation, and sleep	Total: 9–63 Mean of all scores: 1–7	Total: > 36 [53] Mean of all scores: ≥ 4 [53] or ≥ 5 [54]
Fatigue Impact Scale (FIS)	40 items: physical, cognitive, and social	Total: 0–160 Physical: 0–40 Cognitive: 0–40 Social: 0–80	Cut-off not reported [9]
Modified Fatigue Impact Scale (MFIS)	21 items (full-length) or 5 items (abbreviated): physical, cognitive, and psychosocial functioning	21-item version: 0–84 (total) Physical: 0–36 Cognitive: 0–40 Psychosocial: 0–8 5-item version: 0–20	21-item (total): ≥ 38 [55] or ≥ 45 [56] a
Daily Fatigue Impact Scale (D-FIS)	8 items: physical, cognitive, and psychosocial	Total: 0–32	Cut-off not reported [57]
Fatigue Scale for Motor and Cognitive Functions (FSMC)	20 items: Cognition and gait	Total: 20-100 Cognitive: 10-50 Physical: 10-50	Total [58] Mild fatigue: > 42 Moderate fatigue: > 52 Severe fatigue: > 62 Cognitive Mild fatigue: > 21 Moderate fatigue: > 27 Severe fatigue: > 33 Physical Mild fatigue: > 21 Moderate fatigue: > 26 Severe fatigue: > 31

^a^Cut-offs for components and 5-item version unknown

Higher values indicate greater fatigue

##### *Prevalence of Fatigue in Relevant Subgroups:*

Three studies exclusively included patients with CIS, in which fatigue was observed in 18.2 to 46.5% of participants [33, 36, 49]. Excluding the CIS-only studies, the prevalence of fatigue ranged from 35.0 to 97.0% based on both validated and non-validated instruments.

One study specifically examined fatigue in patients with no disability by restricting inclusion to those with Expanded Disability Status Scale [EDSS] scores between 0 and 1.5. Patients with EDSS scores in this range exhibit no or minimal neurological signs of MS. The prevalence of fatigue was estimated to be 35.0% in this sample [20].

The 12 studies that measured fatigue using a validated scale, did not restrict enrolment to CIS only, and did not restrict by level of disability may provide the most reliable and generalizable estimates of fatigue prevalence in the overall MS population. The prevalence of fatigue in these studies ranged from 36.5 to 78.0% [11, 18, 21, 25, 28, 34, 35, 40, 42, 47, 50, 51]. Eight of these studies (describing nine datasets) recruited 300 or more participants, the number required to estimate fatigue prevalence in pwMS with a standard error of ≤5%, assuming that fatigue prevalence was 60%; these studies reported prevalence estimates ranging from 36.5 to 78.0% [11, 25, 34, 35, 40, 42, 48, 50].

##### *Longitudinal Data:*

A single large international study (1401 participants) estimated the prevalence of fatigue at baseline (56.0%) and after 2.5 years (62.5%) using the FSS with fatigue defined as an FSS score ≥ 4 [40].

##### Pediatric and mixed-aged population

Four studies examined fatigue in a pediatric population, reporting a prevalence of 20.0 to 43.0% (Table 2).

Two additional studies included a mix of adult and pediatric patients [17, 19] and another two did not report age-related eligibility criteria [46, 48]. In these four studies, the prevalence of MS-related fatigue ranged from 45.5 to 74.4%.

One study recorded fatigue using the FSS as well as the Neurological Fatigue Index – multiple sclerosis, Brazilian Portuguese version (NFIS-MS/BR) at three time points with three-month intervals; fatigue was defined as FSS ≥28 or NFI-MS/BR ≥30. Of the 26 patients who attended the three interviews, 54.0% of the patients reported persistent fatigue at all three timepoints when measured with the FSS, and 47% with the NFIS-MS/BR [46].

#### Incidence

One Canadian study reported an incidence of fatigue per 100 of 28.9 (95% Confidence Interval [CI]: 23.4, 35.1) at year one after study enrolment, 29.9 (95% CI: 24.5, 35.9) at year two, and overall cumulative incidence of 38.8 (95% CI: 32.7, 45.3) [59].

### Economic burden

A total of nine studies reported the economic burden associated with fatigue in pwMS, in which fatigue was reported categorically (Additional file 3) [11, 13, 19, 60–65]. Most studies were cross-sectional in design (*n* = 7). Sample sizes ranged from 90 to 5173. Most studies were conducted in Europe, North America, and Australasia (*n* = 6). Over half of the studies used the FSS to measure fatigue (*n* = 5) and the most commonly reported outcomes were related to employment (n = 7). All studies were of economic outcomes related to the impact of fatigue on the patient themselves; no records were identified pertaining to the societal or caregiver burden of MS-related fatigue. Two studies examined the relationship between fatigue and direct costs, such as drug costs and physician visits [60, 64]. Seven studies in adult populations reported indirect costs such as employment-related outcomes in relation to fatigue. Results for each study are available in Table 4.

**Table 4:** Results – Economic burden (fatigue assessed as categorical)

Author (year)	Type of Analysis	Sample Size (n)	Cut-off for Fatigue	Outcome	Predictor(s)	Value	95% CI	*p*-value
da Silva (2016)	Multivariate ANOVA	210	MFIS Low impact (39–58), High impact (≥ 59)	Non-DMT costs	EDSS, gender, educational level, **MFIS-BR (cut-off NR)**, MS relapse, any self-reported comorbidities, MS type, and occupation	NR	NR	0.83
Doesburg (2019)	Multiple logistic regression	78	NFI-MS Low (0–10 pts), Middle (11–20), High (21–30)	High work absence	Marital status, relapses in the past year, **NFI-MS (middle vs low)**	OR = 1.41	0.42, 4.76	0.581
					Marital status, relapses in the past year, **NFI-MS (high vs low)**	OR = 15.80	3.00, 83.26	0.001
					Marital status, relapses in the past year, **NFI-MS (high vs middle)**	OR = 11.22	2.13, 59.16	NR
Grytten (2017)	Univariate logistic regression	91	FSS ≥ 4	Unemployment at baseline	**FSS ≥ 4**	OR = 3.03	1.19, 7.71	0.02
	Univariate Cox regression	40		Time to awarding disability pension		HR = 2.03	0.86, 4.78	0.09
Koziarska (2018)	Multivariate logistic regression	150	FSS > 4	Unemployment	**FSS > 4**, EDSS > 3, PQD5, KNS	OR = 2.63	1.02, 6.90	0.046
Lorefice (2018)	Multivariate logistic regression	123	FSS > 4	Unemployment status	Female, age, education, age at onset of MS, disease duration, EDSS, AES-S > 35, BDI-II > 14, **FSS > 4**	OR = 2.10	NR	0.179
McKay (2018)	Generalized estimating equations	340	D-FIS ≥ 5	Hospitalizations	Age, sex, EDSS, **D-FIS ≥ 5**, comorbidity count, HUI pain	adjRR = 1.82	0.86, 3.87	NR
				Physician visit	Age, sex, **D-FIS ≥ 5**, smoker, comorbidity count, HUI pain, HUI cognition	adjRR = 1.06	0.97, 1.17	NR
Razazian (2014)	Pearson’s χ2 test	300	FSS ≥ 5	Medication use	**FSS ≥ 5 vs FSS < 5**	NR	NR	0.002
				Employment status	**FSS ≥ 5 vs FSS < 5**	NR	NR	0.025
Salter (2017)	Multivariable logistic regression	4607	FPS Normal (0), Mild (1, 2), Moderate-to-severe (3–5)	Not working	MS clinical course, age, age at diagnosis, sex, number of comorbidity categories, CPS, **FPS severe (vs. normal)**, HPS, PDDS	OR = 1.93	1.64, 2.26	< 0.0001
		1921		Working < 35 h/week		OR = 1.63	1.04, 2.33	0.0031
		1788		Cut back hrs. Past 6 mos.		OR = 7.19	3.29, 15.70	< 0.0001
		1706		Missed work days past 6 mos.		OR = 4.73	2.67, 8.37	< 0.0001
		1717		Receiving disability benefits		OR = 1.99	1.39, 2.84	0.0005
Weiland (2015)	Binary logistic regression	2133	FSS ≥ 4	FSS ≥ 4	**Work part time**	OR = 1.58	1.24, 2.02	≤ 0.001
					**Stay at home parent/carer**	OR = 19.4	1.36, 2.77	≤ 0.001
					**Unemployed**	OR = 2.15	1.48, 3.11	≤ 0.001
					**Retired due to disability**	OR = 5.54	4.11, 7.47	≤ 0.001
					**Retired due to age**	OR = 1.59	0.94, 2.67	NR
					**Other (inc. student)**	OR = 0.834	0.55,1.27	NR

*Abbreviations*: *adjRR* adjusted rate ratio, *AES-S* Apathy Evaluation Scale, *ANOVA* analysis of variance, *BDI-II* Beck Depression Inventory-Second Edition, *CI* confidence interval, *CPS* Cognition Performance Scale, *D-FIS* daily FIS, *EDSS* Expanded Disability Status Scale, *FIS* Fatigue Impact Scale, *FPS* Fatigue Performance Scale, *FSS* Fatigue Severity Scale, *HPS* Hand function Performance Scale, *HR* hazard ratio, *hrs*. hours, *HUI* Health Utility Index, *KNS* Hope for Success Questionnaire, *MFIS* modified FIS, *MFIS-BR* MFIS Brazilian Portuguese version, *mos*. months, *NFIS-MS/BR* Neurological Fatigue Index – Multiple Sclerosis, Brazilian Portuguese version, *NR* not reported, *OR* odds ratio, *PDDS* Patient Determined Disease Steps, *PQD5* Perceived Deficits Questionnaire 5-items version, *VAS* visual analogue scale

#### Direct costs

Two studies reported the association between fatigue and direct costs. A longitudinal study conducted in Canada examined the association between baseline fatigue (D-FIS ≥ 5) and physician visit and hospitalization rates in pwMS. After adjusting for age, sex, comorbidity count, and other baseline characteristics, no significant associations were found between fatigue and physician visits (adjusted rate ratio = 1.06 [95% CI: 0.97, 1.17]) or hospitalizations (adjusted rate ratio = 1.82 [95% CI: 0.86, 3.87]) [64].

A cross-sectional cost analysis conducted in Brazil reported that higher non–disease-modifying therapy (DMT) direct costs were not associated (*p* = 0.83) with impact of fatigue (MFIS, Brazilian Portuguese version [MFIS-BR], cut-off not reported) after adjusting for disability, gender, educational level, MS relapse, self-reported comorbidities, MS type, and occupation [60].

#### Unemployment

Two European (Poland and Italy) and one North American (USA and Canada) cross-sectional studies assessed whether fatigue was predictive of unemployment in pwMS [62, 63, 65]. Both European studies reported that the odds of being unemployed were higher in pwMS experiencing fatigue (FSS >  4) than in non-fatigued patients after adjustment for patient characteristics such as sex, age, and disability status, although the relationship was only statistically significant in the Polish study (OR = 2.63, 95% CI: 1.02, 6.90 in the Polish study [62]; OR = 2.10, *p* = 0.179 in the Italian study [63]). The North American study also reported that fatigued participants (Fatigue Performance Scale [FPS] 3–5) had statistically significantly higher odds of not working (OR = 1.93; 95% CI: 1.64, 2.26) after adjustment for clinical course, age, and other patient characteristics [65].

One large international cross-sectional study reported that being unemployed was predictive of fatigue (FSS ≥ 4; OR = 2.15; 95% CI: 1.48, 3.11) [11]. Similarly, a Norwegian longitudinal study found fatigue (FSS ≥ 4) was predictive of unemployment at baseline (OR = 3.03 [95% CI: 1.19, 7.71]) [61]. Finally, an Iranian study found that employment status varied between fatigued and non-fatigued participants (*p* = 0.025) [19].

#### Other employment-related outcomes

A North American cross-sectional study found that fatigued participants (FPS 3–5) had statistically significantly higher odds of working less than 35 h per week (OR = 1.63; 95% CI: 1.04, 2.33), cutting back hours in the past 6 months (OR = 7.19; 95% CI: 3.29, 15.70), missing work days in the past 6 months (OR = 4.73; 95% CI: 2.67, 8.37), and receiving disability benefits (OR = 1.99; 95% CI: 1.39, 2.84), after adjustment for clinical course, age, age at diagnosis, sex, comorbidities, cognition, hand function, and disability [65].

A Dutch study found that high fatigue (NFI-MS 21–30) predicted high work absence when compared to low fatigue (NFI-MS 0–10; OR = 15.80; 95% CI: 3.00, 83.26) and intermediate fatigue (NFI-MS 11–20; OR = 11.22; 95% CI: 2.13, 59.16) after adjustment for marital status and relapses in the past year [13].

In contrast to the preceding studies, a Norwegian longitudinal study found fatigue (FSS ≥ 4) did not predict time to awarding disability pension (HR = 2.03; 95% CI: 0.86, 4.78) [61].

#### Supplemental studies

Due to the paucity of studies regarding key economic outcomes such as direct costs and caregiver burden, supplemental screening for studies assessing fatigue as a continuous measure was conducted; 20 additional studies were identified (see Additional file 4). Also included in these 20 are studies in which it was not clear whether fatigue was analyzed as a dichotomous or continuous variable. Most studies reported cross-sectional data (*n* = 17) [66–81]. Most studies were conducted in Europe and North America (*n* = 16) [66–68, 71–79, 81–84], one was conducted in Argentina [69], one in Australia [85], and two did not clearly report the study location [70, 80]. FSS and MFIS were the most commonly used tools to measure fatigue, used in seven studies each. Similar to the categorical studies, the supplemental screening returned a high proportion of studies examining employment-related outcomes (*n* = 18).

Two studies reported data on direct costs of fatigue. A cross-sectional study conducted in Germany found that drug costs and total costs, including indirect costs, drugs, hospital, rehabilitation, etc., were predicted by fatigue (MFIS) after adjusting for depression, disability status, and age [77]. In contrast, a Swedish study found no significant correlation between change in fatigue (FSMC) and change in sickness benefits after 1 year of natalizumab treatment [84].

Eighteen studies reported outcomes pertaining to general indirect costs such as employment/unemployment status and work capacity. One study found that indirect costs, unlike total and drug costs, were not predicted by fatigue [77]. Six studies found an association between fatigue and employment status [67, 69, 74, 80, 82, 86]; conversely, five studies failed to find a statistically significant association [66, 68, 76, 79, 83].

Regarding work capacity outcomes, higher fatigue was associated with sick leave [70] and productivity loss [72, 85] while work capacity was correlated with [73, 81] or impacted by [71, 73, 80, 87] fatigue among other symptoms.

### Humanistic burden

Eleven studies reporting QoL/humanistic burden outcomes were identified through the systematic search (Additional file 3) [11, 12, 17, 18, 88–94]. Most studies were cross-sectional in design (*n* = 10) [11, 12, 17, 18, 89–94], and the sample sizes in all studies ranged from 31 to 2138. Geographically, studies were conducted in Europe (*n* = 6) [12, 17, 18, 88, 91, 92], Brazil (*n* = 3) [89, 93, 94] and Australia (n = 1) [90], with an additional international study where most of the participants reported living in North America, Australasia, and Europe [11]. QoL was assessed in six studies using MS-specific QoL assessment scales (Multiple Sclerosis Quality of Life-54 [MSQOL-54], Multiple Sclerosis International Quality of Life Questionnaire [MusiQoL], and the Functional Assessment of Multiple Sclerosis [FAMS]) [11, 12, 17, 18, 92, 94], while the 36-Item Short Form Survey (SF-36) was applied in four studies [88–91]. One study investigated the humanistic burden of MS-related fatigue by estimating utilities across fatigue levels [93]. Results for each study are available in Table 5.

**Table 5:** Results – Humanistic burden

Author (year)	Type of Analysis	Sample Size	Cut-off for Fatigue	Outcome	Predictor(s)	Value	95% CI	*p*-value
Cioncoloni (2014)	Binary logistic regression	57	FSS ≥ 5	PCS (SF-36) < 40	**FSS ≥ 5**	OR = 11.00	2.97, 40.78	< 0.001
				MCS (SF-36) < 40		OR = 8.64	2.39, 31.28	0.001
Filho (2019) ^a^	Multiple linear regression	31	NR	Vitality (SF-36)	NR; included **FSS (cut-off NR)**	NR	NR	0.006
				Physical Function (SF-36)		NR	NR	0.001
Fricska-Nagy (2016)	Multiple linear regression	428	NR	Overall QoL (MSQOL-54)	BDI-I, EDSS, **cognitive FIS (cut-off NR)**, physical FIS, social FIS	β = 0.094	NR	0.320
					BDI-I, EDSS, cognitive FIS, **physical FIS (cut-off NR)**, social FIS	β = −0.785	NR	0.0001
					BDI-I, EDSS, cognitive FIS, physical FIS, **social FIS (cut-off NR)**	β = − 0.152	NR	0.0001
				Cognitive QoL (MSQOL-54)	BDI-I, EDSS, **cognitive FIS (cut-off NR),** physical FIS, social FIS	β = − 0.550	NR	0.0001
					BDI-I, EDSS, cognitive FIS, **physical FIS (cut-off NR)**, social FIS	β = −0.051	NR	0.475
					BDI-I, EDSS, cognitive FIS, physical FIS, **social FIS (cut-off NR)**	β = −0.130	NR	0.097
				Sexual QoL (MSQOL-54)	BDI-I, EDSS, **cognitive FIS (cut-off NR)**, physical FIS, social FIS	β = −0.249	NR	0.001
					BDI-I, EDSS, cognitive FIS, **physical FIS (cut-off NR),** social FIS	β = 0.008	NR	0.926
					BDI-I, EDSS, cognitive FIS, physical FIS, **social FIS (cut-off NR)**	β = −0.185	NR	0.058
Goksel Karatepe (2011)	Hierarchical regression	79	FSS ≥ 4	Physical health (MSQOL-54)	Disease course, education level, employment status, BDI, EDSS, **FSS ≥ 4**	β = −1.641	−2.99, −0.29	0.018
				Mental health (MSQOL-54)		β = −1.652	−3.26, −0.04	0.045
Gullo (2019)	T-test	62	MFIS, Cognitive > 20, Physical > 23	Physical summary (SF-36)	**Cognitive fatigue** (low vs. high; MFIS)	t = −0.31	NA	0.761
					**Physical fatigue** (low vs. high; MFIS)	t = 3.24	NA	0.002
				Mental summary (SF-36)	**Cognitive fatigue** (low vs. high; MFIS)	t = 4.82	NA	0.001
					**Physical fatigue** (low vs. high; MFIS)	t = 1.90	NA	0.063
Kaya Aygunoglu (2015)	Pearson’s correlation	120	FSS ≥ 4	Physical and mental scores (MSQOL-54)	**FSS**	r = −0.58	NA	< 0.01
Leonavicius (2016)	Multiple linear regression	137	FSS ≥ 4	FSS ≥ 4	Gender, age, residence, education, marital status, professional activity, duration of RRMS, EDSS, DMT, sleep problems, HADS-D, HADS-A, MCS < 50 (SF-36), **PCS < 50 (SF-36**)	OR = 3.82	1.44, 5.54	NR
					Gender, age, residence, education, marital status, professional activity, duration of RRMS, EDSS, DMT, sleep problems, HADS-D, HADS-A, **MCS < 50 (SF-36)**, PCS < 50 (SF-36)	NR	NR	> 0.05
Schmidt (2019)	Multivariate linear regression	254	FSMC ≥43 mild, ≥53 moderate, ≥63 severe	Overall QoL (MusiQoL)	Physical exercise, family status, occupation, CES-D, **FSMC (cut-off NR)**	β = 4.75	1.73, 7.78	0.002
					Family status, occupation, EDSS score, CES-D, **FSMC (cut-off NR)**	β = 3.46	0.51, 6.41	0.022
					CES-D, **FSMC**, EDSS score **(cut-off NR)**	β = 4.98	2.10, 7.87	0.001
					CES-D, **FSMC**, occupation, EDSS score **(cut-off NR)**	β = 4.17	1.29, 7.05	0.005
Takemoto (2015)	Wilcoxon test	210	MFIS-BR Absent: ≤38 points, Low: 39–58 points, High: ≥59 points	Utility score (Brazilian and UK algorithm)	**MFIS-BR** (absent vs. low vs. high)	NR	NA	< 0.001
Taveira (2019)	T-test	39	MFIS ≥38	FAMS	**MFIS** (fatigued vs non-fatigued)	NR	NA	0.001
Weiland (2015) ^b^	Binary logistic regression	2090	FSS ≥ 4	FSS ≥ 4	**Overall HRQoL domain** (MSQOL-54)	OR = 0.94	0.93, 0.94	< 0.001
		1802			**Physical health composite** (MSQOL-54)	OR = 0.91	0.90, 0.92	< 0.001
		2131			**Energy domain** (MSQOL-54)	OR = 0.92	0.92, 0.93	< 0.001
		2047			**Mental health composite** (MSQOL-54)	OR = 0.94	0.93, 0.94	< 0.001

^a^Conference abstract

^b^For every increase of one point in overall MSQOL-54 the odds of clinically significant fatigue reduced by 0.06, 0.09, 0.08, 0.06, respectively

*Abbreviations*: *BDI* Beck Depression Inventory, *BDI-I* Beck Depression Inventory-First Edition, *CES-D* Center for Epidemiological Studies Depression Scale, *CI* confidence interval, *DMT* disease-modifying therapy, *EDSS* Expanded Disability Status Scale, *FAMS* Functional Assessment of Multiple Sclerosis quality of life scale, *FIS* Fatigue Impact Scale, *FSMC* Fatigue Scale for Motor and Cognitive functions, *FSS* Fatigue Severity Scale, *HADS-A* Hospital Anxiety and Depression Scale – Anxiety, *HADS-D* Hospital Anxiety and Depression Scale – Depression, *HRQoL* health-related quality of life, *MCS* mental component summary score of SF-36, *MFIS* Modified Fatigue Impact Scale, *MFIS-BR* MFIS, Brazilian Portuguese version, *MSQOL-54* Multiple Sclerosis Quality of Life-54, *MusiQoL* Multiple Sclerosis International Quality of Life questionnaire, *NR* not reported, *OR* odds ratio, *PCS* physical component summary of SF-36, *QoL* quality of life, *RRMS* relapsing-remitting multiple sclerosis, *SF-36* 36-item Short Form health survey

#### Quality of life

Ten studies investigated the relationship between fatigue and QoL in pwMS [11, 12, 17, 18, 88–92, 94]. Four were European, two South American, two Middle Eastern, one Australian, and one international. The most commonly used scale to report fatigue was the FSS (*n* = 6), followed by the MFIS (*n* = 3), FIS (*n* = 1) and FSMC (n = 1). The SF-36 (*n* = 4) and the MSQOL-54 (*n =* 4) instruments were most often used to measure QoL.

##### The 36-item short form health survey

Four studies used the SF-36 to assess QoL in pwMS. All four studies found a significant association between fatigue and at least one of the subdomains of the SF-36 [88–91].

Two studies (one European and one Brazilian) examined the relationship between fatigue and the SF-36. After adjusting for demographic and socioeconomic variables, duration of RRMS, disease severity, DMT, sleep problems, depression, anxiety and the physical or mental component summary (PCS and MCS respectively) of the SF-36, the European study found higher odds of being fatigued (FSS > 4) with lower PCS scores (< 50) (OR = 3.82 [95% CI: 1.22, 5.54]), but not with lower MCS scores (< 50) (*p* >  0.05) [91]. The Brazilian study found that fatigue (FSS) was associated with a reduction in the physical functioning (*p* = 0.006) and vitality components (*p* = 0.001) of the SF-36 [89].

A second European study explored how fatigue (FSS ≥ 5) relates to physical and mental QoL [88]. This study demonstrated that fatigue was a significant predictor of poorer than average physical QoL (PCS < 40) (OR = 11.00 [95% CI: 2.94, 40.78]) and mental QoL (MCS < 40) (OR = 8.64 [95% CI: 2.39, 31.28]) [88].

An Australian study used the MFIS to measure cognitive (low ≤20, high > 20) and physical fatigue (low ≤23, high > 23) [90]. The study found that physical fatigue was significantly associated with the PCS (t = 3.24, *p* = 0.002) and cognitive fatigue was associated with the MCS (t = 4.82, *p =* 0.002). Cognitive fatigue was not associated with PCS (t = − 0.31, *p* = 0.761) and physical fatigue was not associated with MCS (t = 1.90, *p* = 0.063) [90].

##### Multiple sclerosis quality of Life-54

Four studies used the MSQOL-54 instrument to evaluate QoL [11, 12, 17, 18].

One study examined the relationship between physical, cognitive, and social fatigue measured using FIS with overall QoL, cognitive QoL, and sexual QoL. For each fatigue outcome, the study adjusted for depression, disease severity and the remaining two fatigue types [18]. Physical fatigue was significantly predictive of overall QoL (β = − 0.785, *p* = 0.0001) but not cognitive or sexual QoL.

Three studies used the FSS to measure fatigue. A large international study reported that for a one-point increase in any of the evaluated domains/composites of the MSQOL-54 (i.e., the overall QoL domain, the physical health composite, the energy domain, and the mental health composite), the odds of clinically significant fatigue (FSS ≥ 4) were reduced (OR = 0.94 [95% CI: 0.93, 0.94]; OR = 0.91 [95% CI: 0.90, 0.92]; OR = 0.92 [95% CI: 0.92, 0.93]; OR = 0.94 [95% CI: 0.93, 0.94], respectively) [11]. One study from Turkey, adjusting for disease course, education level, employment status, depression, and disease severity, found that fatigue (FSS ≥ 4) was predictive of physical and mental health based on the MSQOL-54 (β = − 1.641 [95% CI: − 2.99, − 0.29]; β = − 1.652 [95% CI: − 3.26, − 0.04], respectively) [12].

Finally, a second study conducted in Turkey found a strong negative correlation between fatigue (FSS ≥ 4) and the MSQOL-54 physical and mental scores (r = − 0.58, *p* <  0.01) [17].

##### Multiple sclerosis international quality of life

One German study measured QoL using the MusiQoL instrument and the FSMC (cut-off not reported) to measure fatigue [92]. Four analyses were performed with different predictors, and adjusted for different combinations of physical exercise, family status, occupations, depression and disease severity. All found fatigue to be predictive of overall QoL (β ranged from 3.46 to 4.98 and *p* values ranged from 0.001 to 0.022) [92].

##### Functional assessment of multiple sclerosis

One study conducted in Brazil used the FAMS instrument to measure QoL and the MFIS to evaluate fatigue [94]. FAMS score was significantly lower in patients who reported the presence of fatigue (*p* = 0.001, Student’s t-test) [94].

#### Utilities

One Brazilian study used the EQ-5D-3L to investigate the relationship between fatigue and utilities [93]. Fatigue was measured using the MFIS-BR. MS patients were categorized based on the MFIS-BR score into three groups; absent impact, low impact, and high impact of fatigue. The study reported significant differences between the utility scores between the three fatigue groups (*p* <  0.001), indicating a relationship between fatigue and utilities [93].

## Discussion

A comprehensive SLR was conducted following pre-specified inclusion/exclusion criteria in order to understand the burden of MS-related fatigue through a descriptive summary of the published literature, and to identify gaps in current knowledge. Outcomes of interest included prevalence, economic burden, and humanistic burden of MS-related fatigue in patients of any age.

Across studies of adults with sample sizes of > 300 in which a validated fatigue-specific scale was used, and the population was not limited to CIS or non-disabled patients, the prevalence of fatigue ranged from 36.5 to 78.0%. In contrast, when considering all adult studies irrespective of type of MS, disability status, and tool used to estimate fatigue, prevalence ranged from 18.2 to 97.0%.

Nine studies reported data on the economic burden of fatigue in pwMS with fatigue analyzed as a categorical parameter. Of these, seven reported employment-related outcomes such as employment status and sick leave. Of these, all but one study found statistically significant associations between fatigue and the outcomes of interest. Two studies reported data on direct costs and resource utilization, respectively, and found no associations with fatigue.

In contrast, the evidence obtained from the 20 studies included through the supplemental screening for economic outcomes in which fatigue was assessed as a continuous parameter was more heterogeneous. Similar to the categorical studies, most of these records reported data on employment-related outcomes. Of the 11 studies analyzing the impact of fatigue on employment status (e.g. employed vs. unemployed), six found a statistically significant association between the presence of fatigue and unemployment, but no association was found in five other studies. Additionally, eight other studies reported data on the impact of fatigue on outcomes related to work capacity, all of which found a statistically significant association between fatigue and at least one work capacity outcome. One study found an association between fatigue and increased total and drug costs, but no association with indirect costs. Finally, one study found no correlation between fatigue improvement and reduction in sickness benefits.

Over half of the economic studies (including the original and supplementary studies) reported statistically significant associations between fatigue and the economic outcomes evaluated. Of the studies in which the results were not statistically significant, there was a trend for fatigue to be associated with negative impacts on employment-related outcomes.

Eleven studies reported humanistic outcomes, 10 of which were measures of QoL in fatigued and non-fatigued pwMS. A statistically significant association between fatigue and worsening QoL was observed in at least one of the QoL subdomains examined in each of these 10 studies. Only two studies found that physical fatigue was not associated with cognitive or sexual QoL [18, 90]. In the remaining study, statistically significantly lower utilities were observed in pwMS experiencing fatigue [93].

Numerous DMTs are available for the treatment of MS, however outcomes related to fatigue are not consistently reported in trials and it remains uncertain whether some treatments may be more beneficial for alleviating fatigue than others. Non-specific treatments such as amantadine and modafinil have demonstrated a statistically significant impact on fatigue, although the magnitude of benefit is modest at best, with a recent study showing that these treatments are not superior to placebo [95–99].

An important strength of this review is that it adheres to the PRISMA guidelines to ensure best practices for the conduct and reporting of systematic reviews were followed. In particular, a comprehensive literature search was performed and peer-reviewed by experienced information specialists, a detailed grey literature search was conducted, and study selection was performed according to pre-specified criteria. The limitations of this SLR are largely due to the numerous data gaps in the available literature regarding the burden of MS-related fatigue. Very few studies reported on the direct costs associated with fatigue in pwMS. Data were also somewhat sparse for indirect costs; although employment-related outcomes were available, the findings of these studies were not usually translated into monetary values. Therefore, few studies have quantified the indirect financial losses incurred by pwMS experiencing fatigue, their families, and society. The SLR also identified a paucity of longitudinal studies of the impact of fatigue throughout a patient’s life. Moreover, because of the heterogeneity in fatigue scales, methods, and outcome measures between studies, meaningful quantitative synthesis of results across studies was not feasible.

## Conclusions

Clinically relevant fatigue affects a majority of pwMS. There is considerable evidence that the presence of fatigue is associated with poorer employment outcomes, however there was sparse and conflicting evidence as to whether fatigue is associated with greater healthcare costs. There was a lack of evidence regarding the burden of fatigue on caregivers of pwMS, or costs to society more broadly, therefore further study in these areas is required. MS-related fatigue appears to have a negative impact on QoL as measured by both generic HRQoL instruments and MS-specific instruments. It is expected that treatments alleviating fatigue may help mitigate the economic and humanistic burden of this prevalent manifestation of MS.

## Supplementary Information

**Additional file 1: Search strategies.** Full epidemiology search strategy and full economic and quality of life search strategy.

**Additional file 2: List of studies excluded from the SLR.** Full references of excluded studies grouped by reason for exclusion

**Additional file 3: Study and baseline characteristics of included studies.** File includes three tables: 1) Study and baseline characteristics –Epidemiology; 2) Study and baseline characteristics – Economic burden (fatigue assessed as categorical); and 3) Study and baseline characteristics – Humanistic burden.

**Additional file 4: Economic studies reporting fatigue as a linear variable.** File includes two tables: 1) Baseline and study characteristics for economic studies reporting fatigue as a linear variable; and 2) Results of economic studies reporting fatigue as a linear variable.

## Data Availability

The authors declare that the data supporting the findings of this study are available within the article and its supplementary information files. Data are also available from the corresponding author, AK, upon reasonable request.

## Abbreviations

AAN: American Neurology Association
ACTRIMS: Americas Committee for Treatment and Research in Multiple Sclerosis
adjRR: Adjusted rate ratio
AES-S: Apathy Evaluation Scale
AMCP: Academy of Managed Care Pharmacy
ANA: American Neurology Association
ANOVA: Analysis of variance
BDI: Beck Depression Inventory
BDI-I: Beck Depression Inventory-First Edition
BDI-II: Beck Depression Inventory-Second Edition
BOI: Burden of illness
CES-D: Center for Epidemiological Studies Depression Scale
CI: Confidence interval
CIS: Clinically isolated syndrome
CPS: Cognition Performance Scale
D-FIS: Daily Fatigue Impact Scale
DMT: Disease-modifying therapy
EAN: European Academy of Neurology
EBMR: Evidence-Based Medicine Reviews
ECTRIMS: European Committee for Treatment and Research in Multiple Sclerosis
EDSS: Expanded Disability Status Scale
EQ-5D: EuroQoL-5D
FACIT: Functional Assessment of Chronic Illness Therapy
FAMS: Functional Assessment of Multiple Sclerosis
FIS: Fatigue Impact Scale
FPS: Fatigue Performance Scale
FSMC: Fatigue Scale for Motor and Cognitive functions
FSS: Fatigue Severity Score
HADS-A: Hospital Anxiety and Depression Scale – Anxiety
HADS-D: Hospital Anxiety and Depression Scale – Depression
HPS: Hand function Performance Scale
HR: Hazard ratio
HRQoL: Health-related quality of life
HUI: Health Utility Index
ISPOR: International Society for Pharmacoeconomics and Outcomes
KNS: Hope for Success Questionnaire
MCS: Mental component summary score of SF-36
MFIS: Modified Fatigue Impact Scale
MS: Multiple sclerosis
MSQOL-54: Multiple Sclerosis Quality of Life-54
MusiQoL: Multiple Sclerosis International Quality of Life questionnaire
N/A: Not applicable
NFIS-MS: Neurological Fatigue Index – Multiple Sclerosis
NFIS-MS/BR: Neurological Fatigue Index – Multiple Sclerosis, Brazilian Portuguese version
NHS: National Health Service
NMA: Network meta-analysis
NR: Not reported
OR: Odds ratio
PCS: Physical component summary of SF-36
PDDS: Patient Determined Disease Steps
PICOS: Population, intervention, comparator, outcome, and study design
PPMS: Primary progressive MS
PQD5: Perceived Deficits Questionnaire 5-items version
PRESS: Peer Review of Electronic Search Strategies
PRISMA: Preferred Reporting Items for Systematic Reviews and Meta-Analyses
pwMS: People with multiple sclerosis
RCT: Randomized controlled trial
RIS: Radiologically isolated syndrome
RRMS: Relapsing-remitting multiple sclerosis;
SF-36: 36-item Short Form health survey
SLR: Systematic literature review
SPMS: Secondary progressive multiple sclerosis
VAS: Visual analogue scale
