# Supplementary material for: Prevalence and burden of multiple sclerosis-related fatigue: a systematic literature review
Source: BMC Neurol. 2021 Dec 2;21:468. doi: 10.1186/s12883-021-02396-1 (PMC8638268; doi:10.1186/s12883-021-02396-1)
Supplement: Supplementary file 1 — Additional file 1: Search strategies. Full epidemiology search strategy and full economic and quality of life search strategy. [file 12883_2021_2396_MOESM1_ESM.docx]

**Title:** Prevalence and burden of multiple sclerosis-related fatigue: a systematic literature review

**Authors:** Abril Oliva Ramirez, MEpi^1^; Alexander Keenan, MA, MPH^2^; Olivia Kalau^1^; Evelyn Worthington, MSc^1^; Lucas Cohen, MSc^1^; Sumeet Singh, MSc, RPh^1^

^1^EVERSANA, Burlington, Ontario, Canada

^2^Health Economics and Market Access, Janssen Research & Development, LLC, Titusville, NJ, USA

Corresponding author: Alexander Keenan, [AKeenan1@its.jnj.com](mailto:AKeenan1@its.jnj.com), Janssen Scientific Affairs Titusville, NJ, USA

## **Search Strategy**

### **Epidemiology search strategy**

Database: EBM Reviews - Cochrane Central Register of Controlled Trials <December 2019>, EBM Reviews - Cochrane Database of Systematic Reviews <2005 to January 21, 2020>, EBM Reviews - Database of Abstracts of Reviews of Effects <1st Quarter 2016>, EBM Reviews - Health Technology Assessment <4th Quarter 2016>, EBM Reviews - NHS Economic Evaluation Database <1st Quarter 2016>, Embase <1974 to 2020 January 24>, Ovid MEDLINE(R) and Epub Ahead of Print, In-Process & Other Non-Indexed Citations and Daily <1946 to January 24, 2020>

Search Strategy:

--------------------------------------------------------------------------------

1 Demyelinating Autoimmune Diseases, CNS/ (12604)

2 exp Multiple Sclerosis/ (184565)

3 (multiple adj1 (scleros#s or scleroti*)).tw,kf. (193145)

4 ((disseminated or insular or multiplex) adj1 (scleros#s or scleroti*)).tw,kf. (1231)

5 (MS adj10 (scleros#s or scleroti*)).tw,kf. (91882)

6 ((PPMS or PRMS or RPMS or SPMS) adj10 (progressive or relaps* or remit* or scleros#s or scleroti*)).tw,kf. (3616)

7 ((ARMS or RMS or RRMS) adj10 (relaps* or remit* or scleros#s or scleroti*)).tw,kf. (12454)

8 (encephalomyelit* adj2 disseminat*).tw,kf. (4562)

9 or/1-8 [MS] (236471)

10 exp Fatigue/ (257570)

11 Fatigue Syndrome, Chronic/ (10693)

12 fatigu*.tw,kf. (267030)

13 (exertion? or tired* or tiring or weary* or weari*).tw,kf. (113714)

14 (debilit* or devitali* or drows* or enervat* or exhaust* or feeble* or letharg* or lassitude* or listless* or overtired* or sluggish* or somnolen*).tw,kf. (261668)

15 (lack* adj3 (energ* or vitalit*)).tw,kf. (3379)

16 (encephalomyelit* adj2 myalgic).tw,kf. (2027)

17 (infectious adj (mononucleosis* or mono-nucleosis) adj2 syndrome*).tw,kf. (339)

18 royal free disease*.tw,kf. (24)

19 systemic exertion intolerance disease*.tw,kf. (57)

20 or/10-19 [FATIGUE] (724684)

21 9 and 20 [MS + FATIGUE] (14455)

22 exp Animals/ not Humans/ (16982093)

23 21 not 22 [ANIMAL-ONLY REMOVED] (9647)

24 (editorial or news or newspaper article).pt. (1373697)

25 23 not 24 [OPINION PIECES REMOVED] (9558)

26 Demyelinating Autoimmune Diseases, CNS/ep [epidemiology] (149)

27 exp Multiple Sclerosis/ep [epidemiology] (7625)

28 exp Fatigue/ep [epidemiology] (5015)

29 Fatigue Syndrome, Chronic/ep [epidemiology] (1000)

30 exp Epidemiologic Factors/ (4865036)

31 Epidemiological Monitoring/ (8939)

32 epidemiolog*.tw,kf. (878949)

33 Data Collection/ (294356)

34 Incidence/ (651511)

35 (incidenc* or occurren*).tw,kf. (2647510)

36 Prevalence/ (974413)

37 prevalen*.tw,kf. (1807095)

38 Databases, Factual/ (98299)

39 ((clinical or disease* or factual) adj database*).tw,kf. (9654)

40 ((scleros#s or scleroti* or MS) adj3 database*).tw,kf. (2670)

41 ((PPMS or PRMS or RPMS or SPMS or ARMS or RMS or RRMS) adj3 database*).tw,kf. (49)

42 (encephalomyelit* adj3 database*).tw,kf. (3)

43 (fatigu* adj3 database*).tw,kf. (35)

44 Registries/ (157140)

45 (register or registers or registry or registries).tw,kf. (512367)

46 exp Vital Statistics/ (955592)

47 (population statistic* or vital statistic* or vital registration*).tw,kf. (16937)

48 exp Population Surveillance/ (285030)

49 surveil*.tw,kf. (417069)

50 exp "Surveys and Questionnaires"/ (1684438)

51 ((clinical or disease* or health) adj3 (survey* or questionnaire*)).tw,kf. (239464)

52 ((scleros#s or scleroti* or MS) adj3 (survey* or questionnaire*)).tw,kf. (2194)

53 ((PPMS or PRMS or RPMS or SPMS or ARMS or RMS or RRMS) adj3 (survey* or questionnaire*)).tw,kf. (298)

54 (encephalomyelit* adj3 (survey* or questionnaire*)).tw,kf. (10)

55 (fatigu* adj3 (survey* or questionnaire*)).tw,kf. (4012)

56 exp Mortality/ (1422077)

57 Demyelinating Autoimmune Diseases, CNS/mo [mortality] (2)

58 exp Multiple Sclerosis/mo [mortality] (383)

59 exp Fatigue/mo [mortality] (51)

60 Fatigue Syndrome, Chronic/mo [mortality] (5)

61 Morbidity/ (364779)

62 Comorbidity/ (361901)

63 ((scleros#s or scleroti* or MS) adj5 (comorbidit* or co-morbidit* or morbidit* or multimorbid* or multi-morbidit*)).tw,kf. (1608)

64 ((PPMS or PRMS or RPMS or SPMS or ARMS or RMS or RRMS) adj5 (comorbidit* or co-morbidit* or morbidit* or multimorbid* or multi-morbidit*)).tw,kf. (268)

65 (encephalomyelit* adj5 (comorbidit* or co-morbidit* or morbidit* or multimorbid* or multi-morbidit*)).tw,kf. (15)

66 (fatigu* adj5 (comorbidit* or co-morbidit* or morbidit* or multimorbid* or multi-morbidit*)).tw,kf. (991)

67 ((scleros#s or scleroti* or MS) adj5 (frequency or frequencies or number or numbers or rate or rates or statistic*)).tw,kf. (28782)

68 ((PPMS or PRMS or RPMS or SPMS or ARMS or RMS or RRMS) adj5 (frequency or frequencies or number or numbers or rate or rates or statistic*)).tw,kf. (17229)

69 (encephalomyelit* adj5 (frequency or frequencies or number or numbers or rate or rates or statistic*)).tw,kf. (188)

70 (fatigu* adj5 (frequency or frequencies or number or numbers or rate or rates or statistic*)).tw,kf. (10412)

71 or/26-70 [EPI FILTER] (10294041)

72 25 and 71 [MS - FATIGUE - EPIDEMIOLOGY] (3947)

73 limit 72 to yr="2000-current" [Limit not valid in DARE; records were retained] (3730)

74 73 use ppez [MEDLINE RECORDS] (1587)

75 demyelinating disease/ (25760)

76 multiple sclerosis/ (177630)

77 (multiple adj1 (scleros#s or scleroti*)).tw,kw. (194589)

78 ((disseminated or insular or multiplex) adj1 (scleros#s or scleroti*)).tw,kw. (1200)

79 (MS adj10 (scleros#s or scleroti*)).tw,kw. (92043)

80 ((PPMS or PRMS or RPMS or SPMS) adj10 (progressive or relaps* or remit* or scleros#s or scleroti*)).tw,kw. (3634)

81 ((ARMS or RMS or RRMS) adj10 (relaps* or remit* or scleros#s or scleroti*)).tw,kw. (12477)

82 (encephalomyelit* adj2 disseminat*).tw,kw. (4671)

83 or/75-82 [MS] (245597)

84 exp fatigue/ (257570)

85 fatigu*.tw,kw. (272912)

86 (exertion? or tired* or tiring or weary* or weari*).tw,kw. (114170)

87 (debilit* or devitali* or drows* or enervat* or exhaust* or feeble* or letharg* or lassitude* or listless* or overtired* or sluggish* or somnolen*).tw,kw. (265429)

88 (lack* adj3 (energ* or vitalit*)).tw,kw. (3380)

89 (encephalomyelit* adj2 myalgic).tw,kw. (2066)

90 (infectious adj (mononucleosis* or mono-nucleosis) adj2 syndrome*).tw,kw. (339)

91 royal free disease*.tw,kw. (24)

92 systemic exertion intolerance disease*.tw,kw. (68)

93 or/84-92 [FATIGUE] (731712)

94 83 and 93 [MS + FATIGUE] (14658)

95 exp animal/ or exp animal experimentation/ or exp animal model/ or exp animal experiment/ or nonhuman/ or exp vertebrate/ (50392805)

96 exp human/ or exp human experimentation/ or exp human experiment/ (39335869)

97 95 not 96 (11058492)

98 94 not 97 [ANIMAL-ONLY REMOVED] (14453)

99 editorial.pt. (1157559)

100 98 not 99 [OPINION PIECES REMOVED] (14303)

101 demyelinating disease/ep [epidemiology] (319)

102 multiple sclerosis/ep [epidemiology] (7271)

103 exp fatigue/ep [epidemiology] (5015)

104 epidemiological monitoring/ (8939)

105 epidemiological data/ (32036)

106 epidemiolog*.tw,kw. (937137)

107 clinical data repository/ (1129)

108 disease registry/ (13918)

109 incidence/ (651511)

110 familial incidence/ (913)

111 standardized incidence ratio/ (2773)

112 (incidenc* or occurren*).tw,kw. (2652207)

113 exp prevalence/ (1006470)

114 prevalen*.tw,kw. (1811982)

115 factual database/ (101294)

116 ((clinical or disease* or factual) adj database*).tw,kw. (9847)

117 ((scleros#s or scleroti* or MS) adj3 database*).tw,kw. (2675)

118 ((PPMS or PRMS or RPMS or SPMS or ARMS or RMS or RRMS) adj3 database*).tw,kw. (49)

119 (encephalomyelit* adj3 database*).tw,kw. (3)

120 (fatigu* adj3 database*).tw,kw. (36)

121 register/ (113160)

122 (register or registers or registry or registries).tw,kw. (513953)

123 exp population statistics/ (1985412)

124 (population statistic* or vital statistic* or vital registration*).tw,kw. (17241)

125 disease surveillance/ (25864)

126 surveil*.tw,kw. (422580)

127 health survey/ (251655)

128 exp questionnaire/ (1686623)

129 ((clinical or disease* or health) adj3 (survey* or questionnaire*)).tw,kw. (246581)

130 ((scleros#s or scleroti* or MS) adj3 (survey* or questionnaire*)).tw,kw. (2254)

131 ((PPMS or PRMS or RPMS or SPMS or ARMS or RMS or RRMS) adj3 (survey* or questionnaire*)).tw,kw. (299)

132 (encephalomyelit* adj3 (survey* or questionnaire*)).tw,kw. (10)

133 (fatigu* adj3 (survey* or questionnaire*)).tw,kw. (4257)

134 exp mortality rate/ (436438)

135 standardized mortality ratio/ (2782)

136 morbidity/ (364779)

137 comorbidity/ (361901)

138 ((scleros#s or scleroti* or MS) adj5 (comorbidit* or co-morbidit* or morbidit* or multimorbid* or multi-morbidit*)).tw,kw. (1610)

139 ((PPMS or PRMS or RPMS or SPMS or ARMS or RMS or RRMS) adj5 (comorbidit* or co-morbidit* or morbidit* or multimorbid* or multi-morbidit*)).tw,kw. (267)

140 (encephalomyelit* adj5 (comorbidit* or co-morbidit* or morbidit* or multimorbid* or multi-morbidit*)).tw,kw. (15)

141 (fatigu* adj5 (comorbidit* or co-morbidit* or morbidit* or multimorbid* or multi-morbidit*)).tw,kw. (1035)

142 ((scleros#s or scleroti* or MS) adj5 (frequency or frequencies or number or numbers or rate or rates or statistic*)).tw,kw. (28836)

143 ((PPMS or PRMS or RPMS or SPMS or ARMS or RMS or RRMS) adj5 (frequency or frequencies or number or numbers or rate or rates or statistic*)).tw,kw. (17237)

144 (encephalomyelit* adj5 (frequency or frequencies or number or numbers or rate or rates or statistic*)).tw,kw. (184)

145 (fatigu* adj5 (frequency or frequencies or number or numbers or rate or rates or statistic*)).tw,kw. (10762)

146 or/101-145 [EPI FILTER] (8871633)

147 100 and 146 [MS - FATIGUE - EPIDEMIOLOGY] (5642)

148 limit 147 to yr="2000-current" [Limit not valid in DARE; records were retained] (5421)

149 conference abstract.pt. (3701483)

150 148 not 149 (4189)

151 148 and 149 (1232)

152 limit 151 to yr="2018-current" [Limit not valid in DARE; records were retained] (252)

153 150 or 152 [MOST RECENT 2 YRS CONFERENCE ABSTRACTS RETAINED] (4441)

154 153 use oemezd [EMBASE RECORDS] (2188)

155 Demyelinating Autoimmune Diseases, CNS/ (12604)

156 exp Multiple Sclerosis/ (184565)

157 (multiple adj1 (scleros#s or scleroti*)).ti,ab,kw,tw. (194589)

158 ((disseminated or insular or multiplex) adj1 (scleros#s or scleroti*)).ti,ab,kw,tw. (1200)

159 (MS adj10 (scleros#s or scleroti*)).ti,ab,kw,tw. (92043)

160 ((PPMS or PRMS or RPMS or SPMS) adj10 (progressive or relaps* or remit* or scleros#s or scleroti*)).ti,ab,kw,tw. (3634)

161 ((ARMS or RMS or RRMS) adj10 (relaps* or remit* or scleros#s or scleroti*)).ti,ab,kw,tw. (12477)

162 (encephalomyelit* adj2 disseminat*).ti,ab,kw,tw. (4671)

163 or/155-162 [MS] (237217)

164 exp Fatigue/ (257570)

165 Fatigue Syndrome, Chronic/ (10693)

166 fatigu*.ti,ab,kw,tw. (272912)

167 (exertion? or tired* or tiring or weary* or weari*).ti,ab,kw,tw. (114170)

168 (debilit* or devitali* or drows* or enervat* or exhaust* or feeble* or letharg* or lassitude* or listless* or overtired* or sluggish* or somnolen*).ti,ab,kw,tw. (265429)

169 (lack* adj3 (energ* or vitalit*)).ti,ab,kw,tw. (3380)

170 (encephalomyelit* adj2 myalgic).ti,ab,kw,tw. (2066)

171 (infectious adj (mononucleosis* or mono-nucleosis) adj2 syndrome*).ti,ab,kw,tw. (339)

172 royal free disease*.ti,ab,kw,tw. (24)

173 systemic exertion intolerance disease*.ti,ab,kw,tw. (68)

174 or/164-173 [FATIGUE] (732272)

175 163 and 174 [MS + FATIGUE] (14596)

176 Demyelinating Autoimmune Diseases, CNS/ep [epidemiology] (149)

177 exp Multiple Sclerosis/ep [epidemiology] (7625)

178 exp Fatigue/ep [epidemiology] (5015)

179 Fatigue Syndrome, Chronic/ep [epidemiology] (1000)

180 exp Epidemiologic Factors/ (4865036)

181 Epidemiological Monitoring/ (8939)

182 epidemiolog*.ti,ab,kw,tw. (937137)

183 Data Collection/ (294356)

184 Incidence/ (651511)

185 (incidenc* or occurren*).ti,ab,kw,tw. (2652207)

186 Prevalence/ (974413)

187 prevalen*.ti,ab,kw,tw. (1811982)

188 Databases, Factual/ (98299)

189 ((clinical or disease* or factual) adj database*).ti,ab,kw,tw. (9847)

190 ((scleros#s or scleroti* or MS) adj3 database*).ti,ab,kw,tw. (2675)

191 ((PPMS or PRMS or RPMS or SPMS or ARMS or RMS or RRMS) adj3 database*).ti,ab,kw,tw. (49)

192 (encephalomyelit* adj3 database*).ti,ab,kw,tw. (3)

193 (fatigu* adj3 database*).ti,ab,kw,tw. (36)

194 Registries/ (157140)

195 (register or registers or registry or registries).ti,ab,kw,tw. (513953)

196 exp Vital Statistics/ (955592)

197 (population statistic* or vital statistic* or vital registration*).ti,ab,kw,tw. (17241)

198 exp Population Surveillance/ (285030)

199 surveil*.ti,ab,kw,tw. (422580)

200 exp "Surveys and Questionnaires"/ (1684438)

201 ((clinical or disease* or health) adj3 (survey* or questionnaire*)).ti,ab,kw,tw. (246581)

202 ((scleros#s or scleroti* or MS) adj3 (survey* or questionnaire*)).ti,ab,kw,tw. (2254)

203 ((PPMS or PRMS or RPMS or SPMS or ARMS or RMS or RRMS) adj3 (survey* or questionnaire*)).ti,ab,kw,tw. (299)

204 (encephalomyelit* adj3 (survey* or questionnaire*)).ti,ab,kw,tw. (10)

205 (fatigu* adj3 (survey* or questionnaire*)).ti,ab,kw,tw. (4257)

206 exp Mortality/ (1422077)

207 Demyelinating Autoimmune Diseases, CNS/mo [mortality] (2)

208 exp Multiple Sclerosis/mo [mortality] (383)

209 exp Fatigue/mo [mortality] (51)

210 Fatigue Syndrome, Chronic/mo [mortality] (5)

211 Morbidity/ (364779)

212 Comorbidity/ (361901)

213 ((scleros#s or scleroti* or MS) adj5 (comorbidit* or co-morbidit* or morbidit* or multimorbid* or multi-morbidit*)).ti,ab,kw,tw. (1610)

214 ((PPMS or PRMS or RPMS or SPMS or ARMS or RMS or RRMS) adj5 (comorbidit* or co-morbidit* or morbidit* or multimorbid* or multi-morbidit*)).ti,ab,kw,tw. (267)

215 (encephalomyelit* adj5 (comorbidit* or co-morbidit* or morbidit* or multimorbid* or multi-morbidit*)).ti,ab,kw,tw. (15)

216 (fatigu* adj5 (comorbidit* or co-morbidit* or morbidit* or multimorbid* or multi-morbidit*)).ti,ab,kw,tw. (1035)

217 ((scleros#s or scleroti* or MS) adj5 (frequency or frequencies or number or numbers or rate or rates or statistic*)).ti,ab,kw,tw. (28836)

218 ((PPMS or PRMS or RPMS or SPMS or ARMS or RMS or RRMS) adj5 (frequency or frequencies or number or numbers or rate or rates or statistic*)).ti,ab,kw,tw. (17237)

219 (encephalomyelit* adj5 (frequency or frequencies or number or numbers or rate or rates or statistic*)).ti,ab,kw,tw. (184)

220 (fatigu* adj5 (frequency or frequencies or number or numbers or rate or rates or statistic*)).ti,ab,kw,tw. (10762)

221 or/176-220 [EPI FILTER] (10317452)

222 175 and 221 [MS - FATIGUE - EPIDEMIOLOGY] (5992)

223 limit 222 to yr="2000-current" [Limit not valid in DARE; records were retained] (5768)

224 conference abstract.pt. (3701483)

225 223 not 224 (4426)

226 223 and 224 (1342)

227 limit 225 to yr="2018-current" [Limit not valid in DARE; records were retained] (865)

228 225 or 227 [MOST RECENT 2 YRS CONFERENCE ABSTRACTS RETAINED] (4426)

229 228 use coch,cctr,dare,cleed,clhta [COCHRANE RECORDS] (618)

230 74 or 154 or 229 [ALL DATABASES] (4393)

231 remove duplicates from 230 (3172)

232 231 use ppez [MEDLINE UNIQUE RECORDS] (1584)

233 231 use oemezd [EMBASE UNIQUE RECORDS] (1139)

234 231 use coch [DSR UNIQUE RECORDS] (152)

235 231 use cctr [CENTRAL UNIQUE RECORDS] (288)

236 231 use dare [DARE UNIQUE RECORDS] (6)

237 231 use clhta [HTA UNIQUE RECORDS] (0)

238 231 use cleed [NHS EED UNIQUE RECORDS] (3)

***************************

### **Economic and QoL search strategy**

Database: EBM Reviews - Health Technology Assessment <4th Quarter 2016>, EBM Reviews - NHS Economic Evaluation Database <1st Quarter 2016>, Embase <1974 to 2020 January 24>, Ovid MEDLINE(R) and Epub Ahead of Print, In-Process & Other Non-Indexed Citations and Daily <1946 to January 24, 2020>

Search Strategy:

--------------------------------------------------------------------------------

1 Demyelinating Autoimmune Diseases, CNS/ (12601)

2 exp Multiple Sclerosis/ (181491)

3 (multiple adj1 (scleros#s or scleroti*)).tw,kf. (183610)

4 ((disseminated or insular or multiplex) adj1 (scleros#s or scleroti*)).tw,kf. (1218)

5 (MS adj10 (scleros#s or scleroti*)).tw,kf. (87830)

6 ((PPMS or PRMS or RPMS or SPMS) adj10 (progressive or relaps* or remit* or scleros#s or scleroti*)).tw,kf. (3242)

7 ((ARMS or RMS or RRMS) adj10 (relaps* or remit* or scleros#s or scleroti*)).tw,kf. (10413)

8 (encephalomyelit* adj2 disseminat*).tw,kf. (4503)

9 or/1-8 [MS] (225980)

10 exp Fatigue/ (254250)

11 Fatigue Syndrome, Chronic/ (10335)

12 fatigu*.tw,kf. (241792)

13 (exertion? or tired* or tiring or weary* or weari*).tw,kf. (103495)

14 (debilit* or devitali* or drows* or enervat* or exhaust* or feeble* or letharg* or lassitude* or listless* or overtired* or sluggish* or somnolen*).tw,kf. (247951)

15 (lack* adj3 (energ* or vitalit*)).tw,kf. (3131)

16 (encephalomyelit* adj2 myalgic).tw,kf. (1959)

17 (infectious adj (mononucleosis* or mono-nucleosis) adj2 syndrome*).tw,kf. (336)

18 royal free disease*.tw,kf. (22)

19 systemic exertion intolerance disease*.tw,kf. (53)

20 or/10-19 [FATIGUE] (678655)

21 9 and 20 [MS + FATIGUE] (12931)

22 exp Animals/ not Humans/ (16982083)

23 21 not 22 [ANIMAL-ONLY REMOVED] (8123)

24 (editorial or news or newspaper article).pt. (1372734)

25 23 not 24 [OPINION PIECES REMOVED] (8034)

26 Demyelinating Autoimmune Diseases, CNS/ec [economics] (0)

27 exp Multiple Sclerosis/ec [economics] (579)

28 exp Fatigue/ec [economics] (39)

29 Fatigue Syndrome, Chronic/ec [economics] (44)

30 Economics/ (263481)

31 exp "Costs and Cost Analysis"/ (592978)

32 Economics, Nursing/ (34733)

33 Economics, Medical/ (40696)

34 Economics, Pharmaceutical/ (10346)

35 exp Economics, Hospital/ (858145)

36 Economics, Dental/ (34522)

37 exp "Fees and Charges"/ (70696)

38 exp Budgets/ (42387)

39 budget*.ti,ab,kf. (66380)

40 (economic* or cost or costs or costly or costing or price or prices or pricing or pharmacoeconomic* or pharmaco-economic* or expenditure or expenditures or expense or expenses or financial or finance or finances or financed).ti,kf. (476604)

41 (economic* or cost or costs or costly or costing or price or prices or pricing or pharmacoeconomic* or pharmaco-economic* or expenditure or expenditures or expense or expenses or financial or finance or finances or financed).ab. /freq=2 (671349)

42 (cost* adj2 (effective* or utilit* or benefit* or minimi* or analy* or outcome or outcomes)).ab,kf. (371615)

43 (value adj2 (money or monetary)).ti,ab,kf. (5510)

44 exp Models, Economic/ (18291)

45 economic model*.ab,kf. (7640)

46 markov chains/ (20651)

47 markov.ti,ab,kf. (48939)

48 Monte Carlo Method/ (67207)

49 monte carlo.ti,ab,kf. (94721)

50 exp Decision Theory/ (14256)

51 (decision* adj2 (tree* or analy* or model*)).ti,ab,kf. (54940)

52 or/26-51 [ECON FILTER - DIRECT] (2095448)

53 25 and 52 [MS + FATIGUE + ECON FILTER - DIRECT] (471)

54 "Cost of Illness"/ (45816)

55 (cost? adj3 (disease? or illness* or sickness*)).ti,ab,kf. (18447)

56 Absenteeism/ (25912)

57 absentee*.ti,ab,kf. (14185)

58 Presenteeism/ (1427)

59 presentee*.ti,ab,kf. (3418)

60 productivit*.ti,ab,kf. (125820)

61 ((work* or employ* or school* or illness* or sickness*) adj5 (absenc* or absent* or presenc* or present*)).ti,ab,kf. (306406)

62 ((school* or class* or work) adj3 (attend* or miss*)).ti,ab,kf. (35210)

63 ((work* or employ*) adj5 abilit*).ti,ab,kf. (26477)

64 (time adj1 away).ti,ab,kf. (1581)

65 Sick Leave/ (11142)

66 ((sick or medical) adj leave?).ti,ab,kf. (12046)

67 sick day?.ti,ab,kf. (1257)

68 ((wage or wages or income or salary or salaries) adj3 lost).ti,ab,kf. (1503)

69 ((wage or wages or income or salary or salaries) adj3 suppport*).ti,ab,kf. (0)

70 (disabilit* adj3 (support* or payment?)).ti,ab,kf. (3016)

71 Insurance, Health, Reimbursement/ (62693)

72 ((claim? or reimburs*) adj3 (health or insurance)).ti,ab,kf. (21937)

73 ((third-party or 3rd-party) adj2 pay*).ti,ab,kf. (6708)

74 exp Delivery of health Care/ec [economics] (52863)

75 ((healthcare or health care or resource?) adj3 (utili#ation? or utilise? or utilize? or utili#ing)).ti,ab,kf. (80579)

76 Hospitalization/ (460009)

77 (hospitalis* or hospitaliz*).ti,ab,kf. (621682)

78 Length of Stay/ (266065)

79 ((hospital or length) adj2 stay?).ti,ab,kf. (351192)

80 exp Office Visits/ (54070)

81 (visit* adj3 (office? or doctor? or FP or FPs or GP or GPs or MD or MDs or physician? or pcp or pcps or provider? or specialist?)).ti,ab,kf. (47843)

82 or/54-81 [ECON FILTER - INDIRECT] (1922500)

83 25 and 82 [MS + FATIGUE + ECON FILTER - INDIRECT] (438)

84 "Value of Life"/ (135971)

85 Quality of Life/ (640735)

86 quality of life.ti,kf. (181480)

87 ((instrument or instruments) adj3 quality of life).ab. (7825)

88 Quality-Adjusted Life Years/ (40692)

89 quality adjusted life.ti,ab,kf. (31419)

90 (qaly* or qald* or qale* or qtime* or life year or life years).ti,ab,kf. (51516)

91 disability adjusted life.ti,ab,kf. (7035)

92 daly*.ti,ab,kf. (6721)

93 (sf36 or sf 36 or short form 36 or shortform 36 or short form36 or shortform36 or sf thirtysix or sfthirtysix or sfthirty six or sf thirty six or shortform thirtysix or shortform thirty six or short form thirtysix or short form thirty six).ti,ab,kf. (65319)

94 (sf6 or sf 6 or short form 6 or shortform 6 or sf six or sfsix or shortform six or short form six or shortform6 or short form6).ti,ab,kf. (4424)

95 (sf8 or sf 8 or sf eight or sfeight or shortform 8 or shortform 8 or shortform8 or short form8 or shortform eight or short form eight).ti,ab,kf. (1256)

96 (sf12 or sf 12 or short form 12 or shortform 12 or short form12 or shortform12 or sf twelve or sftwelve or shortform twelve or short form twelve).ti,ab,kf. (14685)

97 (sf16 or sf 16 or short form 16 or shortform 16 or short form16 or shortform16 or sf sixteen or sfsixteen or shortform sixteen or short form sixteen).ti,ab,kf. (88)

98 (sf20 or sf 20 or short form 20 or shortform 20 or short form20 or shortform20 or sf twenty or sftwenty or shortform twenty or short form twenty).ti,ab,kf. (843)

99 (hql or hqol or h qol or hrqol or hr qol).ti,ab,kf. (43548)

100 (hye or hyes).ti,ab,kf. (200)

101 (health* adj2 year* adj2 equivalent*).ti,ab,kf. (100)

102 (pqol or qls).ti,ab,kf. (1005)

103 (quality of wellbeing or quality of well being or index of wellbeing or index of well being or qwb).ti,ab,kf. (1261)

104 nottingham health profile*.ti,ab,kf. (2659)

105 sickness impact profile.ti,ab,kf. (2277)

106 exp health status indicators/ (325648)

107 (health adj3 (utilit* or status)).ti,ab,kf. (158804)

108 (utilit* adj3 (valu* or measur* or health or life or estimat* or elicit* or disease or score* or weight)).ti,ab,kf. (30435)

109 (preference* adj3 (valu* or measur* or health or life or estimat* or elicit* or disease or score* or instrument or instruments)).ti,ab,kf. (24017)

110 disutilit*.ti,ab,kf. (1345)

111 rosser.ti,ab,kf. (214)

112 willingness to pay.ti,ab,kf. (13723)

113 standard gamble*.ti,ab,kf. (1932)

114 (time trade off or time tradeoff).ti,ab,kf. (3344)

115 tto.ti,ab,kf. (2637)

116 (hui or hui1 or hui2 or hui3).ti,ab,kf. (3658)

117 (eq or euroqol or euro qol or eq5d or eq 5d or euroqual or euro qual).ti,ab,kf. (40688)

118 duke health profile.ti,ab,kf. (196)

119 functional status questionnaire.ti,ab,kf. (280)

120 dartmouth coop functional health assessment*.ti,ab,kf. (26)

121 ("9-Hole Peg Test" or 9-HPT).tw,kf. (989)

122 (Multiple Sclerosis Functional Composite or MSFC).ti,ab,kf. (1173)

123 (Multiple Sclerosis Quality of Life-54 or MSQOL-54).ti,ab,kf. (567)

124 (Multiple Sclerosis Quality of Life Inventory or MSQLI).ti,ab,kf. (73)

125 (Functional Assessment of Chronic Illness Therapy-Spiritual Well Being Scale or FACIT-SP).ti,ab,kf. (526)

126 (Functional Systems Scores or FSS or Expanded Disability Status Scale or EDSS).ti,ab,kw. (26298)

127 (Fatigue Symptom Inventory or FSI).ti,ab,kf. (2467)

128 (Modified Fatigue Impact Scale or MFIS).ti,ab,kf. (1629)

129 (Multidimensional Fatigue Symptom Inventory or MFSI).ti,ab,kf. (240)

130 or/84-129 [Health Utilities/QoL Filter] (1365807)

131 25 and 130 (3298)

132 53 or 83 or 131 [MS + FATIGUE + ECOMOMICS/HU/QoL] (3662)

133 limit 132 to yr="2000-current" (3499)

134 133 use ppez (1627)

135 demyelinating disease/ (25681)

136 multiple sclerosis/ (175276)

137 (multiple adj1 (scleros#s or scleroti*)).tw,kw. (184697)

138 ((disseminated or insular or multiplex) adj1 (scleros#s or scleroti*)).tw,kw. (1187)

139 (MS adj10 (scleros#s or scleroti*)).tw,kw. (87988)

140 ((PPMS or PRMS or RPMS or SPMS) adj10 (progressive or relaps* or remit* or scleros#s or scleroti*)).tw,kw. (3260)

141 ((ARMS or RMS or RRMS) adj10 (relaps* or remit* or scleros#s or scleroti*)).tw,kw. (10436)

142 (encephalomyelit* adj2 disseminat*).tw,kw. (4602)

143 or/135-142 [MS] (234806)

144 exp fatigue/ (254250)

145 fatigu*.tw,kw. (244058)

146 (exertion? or tired* or tiring or weary* or weari*).tw,kw. (103946)

147 (debilit* or devitali* or drows* or enervat* or exhaust* or feeble* or letharg* or lassitude* or listless* or overtired* or sluggish* or somnolen*).tw,kw. (248548)

148 (lack* adj3 (energ* or vitalit*)).tw,kw. (3132)

149 (encephalomyelit* adj2 myalgic).tw,kw. (1998)

150 (infectious adj (mononucleosis* or mono-nucleosis) adj2 syndrome*).tw,kw. (336)

151 royal free disease*.tw,kw. (22)

152 systemic exertion intolerance disease*.tw,kw. (64)

153 or/144-152 [FATIGUE] (680005)

154 143 and 153 [MS + FATIGUE] (13029)

155 exp animal/ or exp animal experimentation/ or exp animal model/ or exp animal experiment/ or nonhuman/ or exp vertebrate/ (49819920)

156 exp human/ or exp human experimentation/ or exp human experiment/ (38763002)

157 155 not 156 (11058474)

158 154 not 157 [ANIMAL-ONLY REMOVED] (12824)

159 editorial.pt. (1157105)

160 158 not 159 [OPINION PIECES REMOVED] (12674)

161 economics/ (263481)

162 cost/ (108571)

163 exp health economics/ (832849)

164 budget/ (39941)

165 budget*.ti,ab,kw. (66752)

166 (economic* or cost or costs or costly or costing or price or prices or pricing or pharmacoeconomic* or pharmaco-economic* or expenditure or expenditures or expense or expenses or financial or finance or finances or financed).ti,kw. (499324)

167 (economic* or cost or costs or costly or costing or price or prices or pricing or pharmacoeconomic* or pharmaco-economic* or expenditure or expenditures or expense or expenses or financial or finance or finances or financed).ab. /freq=2 (671349)

168 (cost* adj2 (effective* or utilit* or benefit* or minimi* or analy* or outcome or outcomes)).ab,kw. (375848)

169 (value adj2 (money or monetary)).ti,ab,kw. (5520)

170 statistical model/ (248607)

171 economic model*.ab,kw. (7888)

172 probability/ (156740)

173 markov chains/ (20651)

174 markov.ti,ab,kw. (49656)

175 monte carlo method/ (67207)

176 monte carlo.ti,ab,kw. (95916)

177 decision theory/ (2643)

178 decision tree/ (23802)

179 (decision* adj2 (tree* or analy* or model*)).ti,ab,kw. (55776)

180 or/161-179 [ECON FILTER - DIRECT] (2330196)

181 160 and 180 [MS + FATIGUE + ECON FILTER - DIRECT] (748)

182 "cost of illness"/ (45816)

183 (cost? adj3 (disease? or illness* or sickness*)).ti,ab,kw. (19106)

184 hospitalization cost/ (6524)

185 absenteeism/ (25912)

186 absentee*.ti,ab,kw. (14429)

187 presenteeism/ (1427)

188 presentee*.ti,ab,kw. (3497)

189 productivity/ (52017)

190 productivit*.ti,ab,kw. (126549)

191 ((work* or employ* or school* or illness* or sickness*) adj5 (absenc* or absent* or presenc* or present*)).ti,ab,kw. (306548)

192 ((school* or class* or work) adj3 (attend* or miss*)).ti,ab,kw. (35261)

193 ((work* or employ*) adj5 abilit*).ti,ab,kw. (26545)

194 (time adj1 away).ti,ab,kw. (1584)

195 medical leave/ (6485)

196 ((sick or medical) adj leave?).ti,ab,kw. (12216)

197 sick day?.ti,ab,kw. (1263)

198 ((wage or wages or income or salary or salaries) adj3 lost).ti,ab,kw. (1506)

199 ((wage or wages or income or salary or salaries) adj3 suppport*).ti,ab,kw. (0)

200 (disabilit* adj3 (support* or payment?)).ti,ab,kw. (3046)

201 reimbursement/ (56064)

202 ((claim? or reimburs*) adj3 (health or insurance)).ti,ab,kw. (22100)

203 ((third-party or 3rd-party) adj2 pay*).ti,ab,kw. (6725)

204 ((healthcare or health care or resource?) adj3 (utili#ation? or utilise? or utilize? or utili#ing)).ti,ab,kw. (81260)

205 Hospitalization/ (460009)

206 (hospitalis* or hospitaliz*).ti,ab,kw. (623240)

207 length of stay/ (266065)

208 ((hospital or length) adj2 stay?).ti,ab,kw. (351595)

209 (visit* adj3 (office? or doctor? or FP or FPs or GP or GPs or MD or MDs or physician? or pcp or pcps or provider? or specialist?)).ti,ab,kw. (47914)

210 or/182-209 [ECON FILTER - INDIRECT] (1857195)

211 160 and 210 [MS + FATIGUE + ECON FILTER - INDIRECT] (667)

212 socioeconomics/ (136593)

213 exp quality of life/ (661931)

214 quality of life.ti,kw. (211937)

215 ((instrument or instruments) adj3 quality of life).ab. (7825)

216 quality-adjusted life year/ (40692)

217 quality adjusted life.ti,ab,kw. (31574)

218 (qaly* or qald* or qale* or qtime* or life year or life years).ti,ab,kw. (51856)

219 disability adjusted life.ti,ab,kw. (7047)

220 daly*.ti,ab,kw. (6828)

221 (sf36 or sf 36 or short form 36 or shortform 36 or short form36 or shortform36 or sf thirtysix or sfthirtysix or sfthirty six or sf thirty six or shortform thirtysix or shortform thirty six or short form thirtysix or short form thirty six).ti,ab,kw. (65596)

222 (sf6 or sf 6 or short form 6 or shortform 6 or sf six or sfsix or shortform six or short form six or shortform6 or short form6).ti,ab,kw. (4436)

223 (sf8 or sf 8 or sf eight or sfeight or shortform 8 or shortform 8 or shortform8 or short form8 or shortform eight or short form eight).ti,ab,kw. (1259)

224 (sf12 or sf 12 or short form 12 or shortform 12 or short form12 or shortform12 or sf twelve or sftwelve or shortform twelve or short form twelve).ti,ab,kw. (14742)

225 (sf16 or sf 16 or short form 16 or shortform 16 or short form16 or shortform16 or sf sixteen or sfsixteen or shortform sixteen or short form sixteen).ti,ab,kw. (88)

226 (sf20 or sf 20 or short form 20 or shortform 20 or short form20 or shortform20 or sf twenty or sftwenty or shortform twenty or short form twenty).ti,ab,kw. (843)

227 (hql or hqol or h qol or hrqol or hr qol).ti,ab,kw. (43742)

228 (hye or hyes).ti,ab,kw. (204)

229 (health* adj2 year* adj2 equivalent*).ti,ab,kw. (103)

230 (pqol or qls).ti,ab,kw. (1007)

231 (quality of wellbeing or quality of well being or index of wellbeing or index of well being or qwb).ti,ab,kw. (1269)

232 nottingham health profile/ (480)

233 nottingham health profile*.ti,ab,kw. (2671)

234 sickness impact profile/ (9451)

235 sickness impact profile.ti,ab,kw. (2316)

236 health status indicator/ (26119)

237 (health adj3 (utilit* or status)).ti,ab,kw. (160279)

238 (utilit* adj3 (valu* or measur* or health or life or estimat* or elicit* or disease or score* or weight)).ti,ab,kw. (30563)

239 (preference* adj3 (valu* or measur* or health or life or estimat* or elicit* or disease or score* or instrument or instruments)).ti,ab,kw. (24089)

240 disutilit*.ti,ab,kw. (1348)

241 rosser.ti,ab,kw. (215)

242 willingness to pay.ti,ab,kw. (13867)

243 standard gamble*.ti,ab,kw. (1951)

244 (time trade off or time tradeoff).ti,ab,kw. (3375)

245 tto.ti,ab,kw. (2646)

246 (hui or hui1 or hui2 or hui3).ti,ab,kw. (3673)

247 (eq or euroqol or euro qol or eq5d or eq 5d or euroqual or euro qual).ti,ab,kw. (40750)

248 duke health profile.ti,ab,kw. (196)

249 expanded disability status scale/ (12620)

250 exp functional status assessment/ (143997)

251 functional status questionnaire.ti,ab,kw. (280)

252 dartmouth coop functional health assessment*.ti,ab,kw. (26)

253 ("9-Hole Peg Test" or 9-HPT).ti,ab,kw. (989)

254 (Multiple Sclerosis Functional Composite or MSFC).ti,ab,kw. (1187)

255 (Multiple Sclerosis Quality of Life-54 or MSQOL-54).ti,ab,kw. (567)

256 (Multiple Sclerosis Quality of Life Inventory or MSQLI).ti,ab,kw. (73)

257 (Functional Assessment of Chronic Illness Therapy-Spiritual Well Being Scale or FACIT-SP).ti,ab,kw. (528)

258 (Functional Systems Scores or FSS or Expanded Disability Status Scale or EDSS).ti,ab,kw. (26298)

259 (Fatigue Symptom Inventory or FSI).ti,ab,kw. (2488)

260 (Modified Fatigue Impact Scale or MFIS).ti,ab,kw. (1635)

261 (Multidimensional Fatigue Symptom Inventory or MFSI).ti,ab,kw. (240)

262 or/212-261 [Health Utilities/QoL Filter] (1203922)

263 160 and 262 (5660)

264 181 or 211 or 263 [MS + FATIGUE + ECOMOMICS/HU/QoL] (6247)

265 limit 264 to yr="2000-current" (6096)

266 conference abstract.pt. (3684740)

267 265 not 266 (4220)

268 265 and 266 (1876)

269 limit 268 to yr="2018-current" (325)

270 267 or 269 [MOST RECENT 2 YEARS CONFERENCE ABSTRACTS RETAINED] (4545)

271 270 use oemezd [EMBASE RECORDS] (3041)

272 Demyelinating Autoimmune Diseases, CNS/ (12601)

273 exp Multiple Sclerosis/ (181491)

274 (multiple adj1 (scleros#s or scleroti*)).tw. (182123)

275 ((disseminated or insular or multiplex) adj1 (scleros#s or scleroti*)).tw. (1173)

276 (MS adj10 (scleros#s or scleroti*)).tw. (87704)

277 ((PPMS or PRMS or RPMS or SPMS) adj10 (progressive or relaps* or remit* or scleros#s or scleroti*)).tw. (3236)

278 ((ARMS or RMS or RRMS) adj10 (relaps* or remit* or scleros#s or scleroti*)).tw. (10395)

279 (encephalomyelit* adj2 disseminat*).tw. (4460)

280 or/272-279 [MS] (225249)

281 exp Fatigue/ (254250)

282 Fatigue Syndrome, Chronic/ (10335)

283 fatigu*.tw. (240276)

284 (exertion? or tired* or tiring or weary* or weari*).tw. (101220)

285 (debilit* or devitali* or drows* or enervat* or exhaust* or feeble* or letharg* or lassitude* or listless* or overtired* or sluggish* or somnolen*).tw. (247163)

286 (lack* adj3 (energ* or vitalit*)).tw. (3129)

287 (encephalomyelit* adj2 myalgic).tw. (1905)

288 (infectious adj (mononucleosis* or mono-nucleosis) adj2 syndrome*).tw. (334)

289 royal free disease*.tw. (22)

290 systemic exertion intolerance disease*.tw. (42)

291 or/281-290 [FATIGUE] (674920)

292 280 and 291 [MS + FATIGUE] (12898)

293 limit 292 to yr="2000-current" (12078)

294 293 use cleed,clhta [COCHRANE DATABASES] (9)

295 134 or 271 or 294 [ALL DATABASES] (4677)

296 remove duplicates from 295 (3258)

297 296 use ppez [MEDLINE UNIQUE RECORDS] (1625)

298 296 use oemezd [EMBASE UNIQUE RECORDS] (1624)

299 296 use clhta [HTA UNIQUE RECORDS] (5)

300 296 use cleed [NHS EED UNIQUE RECORDS] (4)

***************************
