# Supplementary material for: Prevalence and burden of multiple sclerosis-related fatigue: a systematic literature review
Source: BMC Neurol. 2021 Dec 2;21:468. doi: 10.1186/s12883-021-02396-1 (PMC8638268; doi:10.1186/s12883-021-02396-1)
Supplement: Supplementary file 2 — Additional file 2: List of studies excluded from the SLR. Full references of excluded studies grouped by reason for exclusion [file 12883_2021_2396_MOESM2_ESM.docx]

**Title:** Prevalence and burden of multiple sclerosis-related fatigue: a systematic literature review

**Authors:** Abril Oliva Ramirez, MEpi^1^; Alexander Keenan, MA, MPH^2^; Olivia Kalau^1^; Evelyn Worthington, MSc^1^; Lucas Cohen, MSc^1^; Sumeet Singh, MSc, RPh^1^

^1^EVERSANA, Burlington, Ontario, Canada

^2^Health Economics and Market Access, Janssen Research & Development, LLC, Titusville, NJ, USA

Corresponding author: Alexander Keenan, [AKeenan1@its.jnj.com](mailto:AKeenan1@its.jnj.com), Janssen Scientific Affairs Titusville, NJ, USA

## **List of studies excluded from the SLR**

**Non-English**

Astudilla P, Machado R, Peralles S, Striebel VLW (2011) Association between fatigue and quality of life in multiple sclerosis patients: A literature review. Revista Neurociencias 19 (3): 525.

Baggio BF, Teles RA, Renosto A, Alvarenga LFC (2011) Epidemiologic profile of individuals with multiple sclerosis from a pool of reference. Revista Neurociencias 19 (3): 458.

**Population**

Adamopoulou F, Alikari V, Zyga S, Tsironi M, Tzavella F et al. (2019) The Effect of Fatigue and Pain Self- Efficacy on Health-Related Quality of Life Among Patients with Multiple Sclerosis. Mater socio-med.. 31 (1): 40.

Akaishi T, Nakashima I, Misu T, Fujihara K, Aoki M (2015) Depressive state and chronic fatigue in multiple sclerosis and neuromyelitis optica. Journal of neuroimmunology 283 70.

Backus D, Manella C, Bender A, Sweatman M (2016) Impact of Massage Therapy on Fatigue, Pain, and Spasticity in People with Multiple Sclerosis: a Pilot Study. Int J Ther Massage Bodywork 9 (4): 4.

Berger T, Kobelt G, Berg J, Capsa D, Gannedahl M et al. (2017) New insights into the burden and costs of multiple sclerosis in Europe: Results for Austria. Multiple Sclerosis 23 (2_suppl): 17.

Bermel R, Mowry EM, Krupp L, Jones S, Naismith R et al. (2018) The multiple sclerosis partners advancing technology and health solutions (MS paths) patient cohort. Neurology Conference: (15 Supplement 1):

Berriozabalgoitia R, Otxoa E, Esain I, Bidaurrazaga I, Espin A et al. (2019) Walking and fatigue correlate with quality of life. Multiple Sclerosis Journal Conference: (7): 1072.

Bueno AM, Sayao AL, Yousefi M, Devonshire V, Traboulsee A et al. (2015) Health-related quality of life in patients with longstanding 'benign multiple sclerosis'. Multiple Sclerosis and Related Disorders 4 (1): 31.

Canzonieri AM, Simandi TM, Fernandes DGDO (2018) Epidemiological data of patients with multiple sclerosis. Multiple Sclerosis Journal Conference: (2): NP3.

Carroll CA, Fairman KA, Lage MJ (2014) Updated cost-of-care estimates for commercially insured patients with multiple sclerosis: retrospective observational analysis of medical and pharmacy claims data. BMC Health Services Research [electronic resource] 14 286.

Castelli L, Luca FD, Marchetti MR, Sellitto G, Fanelli F et al. (2016) The dual task-cost of standing balance affects quality of life in mildly disabled MS people. Neurological Sciences 37 (5): 673.

Cederberg KL, Jeng B, Sasaki JE, Braley TJ, Walters AS et al. (2019) Restless legs syndrome and health-related quality of life in adults with multiple sclerosis. Sleep Conference: (Supplement 1): A264.

Chanthavinout K, Miranda DS, Kelley NW, Bucar D, Capkun G (2018) Impact of fatigue on resource utilization and healthcare costs in autoimmune (Ai) Disease. Value in Health Conference: (Supplement 3): S462.

Chen K, Fan Y, Hu R, Yang T, Li K (2013) Impact of depression, fatigue and disability on quality of life in Chinese patients with multiple sclerosis. Stress health 29 (2): 108.

Ciampi E, Uribe-San-Martin R, Vasquez M, Ruiz-Tagle A, Labbe T et al. (2018) Relationship between Social Cognition and traditional cognitive impairment in Progressive Multiple Sclerosis and possible implicated neuroanatomical regions. Multiple Sclerosis and Related Disorders 20 122.

Cochrane GD, Rizvi S, Abrantes AM, Crabtree B, Cahill J et al. (2015) The association between fatigue and apathy in patients with either Parkinson's disease or multiple sclerosis. Parkinsonism Relat Disord 21 (9): 1093.

Cosio D, Jin L, Siddique J, Mohr DC (2011) The effect of telephone-administered cognitive-behavioral therapy on quality of life among patients with multiple sclerosis. Annals of Behavioral Medicine 41 (2): 227.

Disanto G, Zecca C, MacLachlan S, Sacco R, Handunnetthi L et al. (2018) Prodromal symptoms of multiple sclerosis in primary care. Annals of Neurology 83 (6): 1162.

Dubois B, Kobelt G, Berg J, Capsa D, Gannedahl M et al. (2017) New insights into the burden and costs of multiple sclerosis in Europe: Results for Belgium. Multiple Sclerosis 23 (2_suppl): 29.

Dymecka J, Bidzan M (2018) Biomedical Variables and Adaptation to Disease and Health-Related Quality of Life in Polish Patients with MS. 15 (12):

Enns MW, Bernstein CN, Kroeker K, Graff L, Walker JR et al. (2018) The association of fatigue, pain, depression and anxiety with work and activity impairment in immune mediated inflammatory diseases. PLoS One 13 (6): e0198975, 0192018.

Ertekin O, Ozakbas S, Idiman E, Algun ZC (2012) Quality of life, fatigue and balance improvements after home-based exercise program in multiple sclerosis patients. Noropsikiyatri Arsivi 49 (1): 33.

Ertekin O, Ozakbas S, Idiman E, Algun ZC (2013) The effects of low and severe disability on walking abilities and quality of life in multiple sclerosis patients: 6-month follow-up study. Noropsikiyatri Arsivi 50 (1): 23.

Fidao ALR, Livera AMD, Weiland TJ, Jelinek GA, Brown CR et al. (2019) Depression but not physical activity mediates the fatigue-mental quality of life relationship in multiple sclerosis. Multiple Sclerosis Journal Conference: (Supplement 1): 73.

Fragoso YD, Finkelsztejn A, Giacomo MC, Russo L, Cruz WS (2010) The effect of multiple sclerosis on the professional life of a group of Brazilian patients. Arquivos de Neuro-Psiquiatria 68 (6): 914.

Garg H, Bush S, Gappmaier E (2016) Associations Between Fatigue and Disability, Functional Mobility, Depression, and Quality of Life in People with Multiple Sclerosis. 18 (2): 71.

Gervasoni E, Giovanni RD, Anastasi D, Brichetto G, Carpinella I et al. (2019) Identification of functional disorders in early diagnosed People with Multiple Sclerosis: A window of opportunity? Multiple Sclerosis Journal Conference: (7): 1047.

Gomes OM, Khillare SD (2018) Fatigue and quality of life in persons with multiple sclerosis-a correlative study. Neurorehabilitation and Neural Repair Conference: (4-5): 434.

Goodwin E, Green C, Hawton A (2019) What Difference Does It Make? A Comparison of Health State Preferences Elicited From the General Population and From People With Multiple Sclerosis. Value in Health

Hiele Kvd, Spliethoff-Kamminga NG, Ruimschotel RP, Middelkoop HA, Visser LH (2012) Daily hassles reported by Dutch multiple sclerosis patients. Journal of the Neurological Sciences 320 (1-2): 85.

Karampampa K, Gustavsson A, Munster ETv, Hupperts RM, Sanders EA et al. (2013) Treatment experience, burden, and unmet needs (TRIBUNE) in Multiple Sclerosis study: the costs and utilities of MS patients in The Netherlands. Journal of Medical Economics 16 (7): 939.

Kessel KV, Babbage DR, Reay N, Miner-Williams WM, Kersten P (2017) Mobile Technology Use by People Experiencing Multiple Sclerosis Fatigue: Survey Methodology. JMIR Mhealth Uhealth 5 (2): e6.

Kister I, Bacon T, Wang L, Cutter G (2019) Longitudinal study of symptom botheration in multiple sclerosis patients. Neurology Conference: (15 Supplement 1):

Kister I, Bacon TE, Chamot E, Salter AR, Cutter GR et al. (2013) Natural history of multiple sclerosis symptoms. 15 (3): 146.

Kobelt G, Langdon D, Jonsson L (2019) The effect of self-assessed fatigue and subjective cognitive impairment on work capacity: The case of multiple sclerosis. Multiple Sclerosis 25 (5): 740.

Kuttuvajeyaram V, Ranganathan L, Manickavasagam J, Chandramouleeswaran V, Sarala G et al. (2018) A study on assessment of fatigue in multiple sclerosis. Neurorehabilitation and Neural Repair Conference: (4-5): 535.

Laabidi K, Derbali H, Riahi A, Messelmani M, Mansour M et al. (2019) Fatigue in multiple sclerosis: The involvement of sleep disturbances and urinary disorders. Journal of the Neurological Sciences Conference: (WCN 2019). United Arab Emirates. 405 (Supplement): 289.

Lin PJ, Saret CJ, Neumann PJ, Sandberg EA, Cohen JT (2016) Assessing the Value of Treatment to Address Various Symptoms Associated with Multiple Sclerosis: Results from a Contingent Valuation Study. Pharmacoeconomics 34 (12): 1255.

Lukoschek C, Sterr A, Claros-Salinas D, Gutler R, Dettmers C (2015) Fatigue in Multiple Sclerosis Compared to Stroke. Frontiers in Neurology 6 116.

Mahmoud MB, Derbali H, Riahi A, Azzouz D, Zaouali J et al. (2019) Fatigue in multiple sclerosis: A Tunisian hospital cohort. Journal of the Neurological Sciences Conference: (WCN 2019). United Arab Emirates. 405 (Supplement): 281.

Marrie RA, Cutter GR, Tyry T, Cofield SS, Fox R et al. (2017) Upper limb impairment is associated with use of assistive devices and unemployment in multiple sclerosis. Multiple Sclerosis and Related Disorders 13 87.

McKee MG, Grossman EF, Reynard AK, Bolwell G, Schneeberger DL et al. (2014) Biofeedback training in patients with multiple sclerosis. Psychosomatic Medicine 76 (3): A.

Miller D, Mowry E, Planchon S, Moore CD, Bermel R (2018) Association between Neuro-QoL scale scores and employment status in MS PATHS (Multiple Sclerosis Partners Advancing Technology and Health Solutions) patients. Quality of Life Research Conference: (Supplement 1): S54.

Minar M, Petrlenicova D, Valkovic P (2016) Restless legs syndrome in multiple sclerosis decreases health-related quality of life. Activitas Nervosa Superior Rediviva 58 (1): 19.

Morrow S, Bhatty D, Blair M, Mehta S, Gill S (2018) Predictors of vocational status among persons with multiple sclerosis. Neurology Conference: (15 Supplement 1):

Munsell M, Frean M, Menzin J, Phillips AL (2017) Development and validation of a claims-based measure as an indicator for disease status in patients with multiple sclerosis treated with disease-modifying drugs. BMC Neurology 17 (1): 106.

Nagaraj K, Taly AB, Gupta A, Prasad C, Christopher R (2013) Prevalence of fatigue in patients with multiple sclerosis and its effect on the quality of life. J Neurosci Rural Pract 4 (3): 278.

Nyland M, Naess H, Lode K, Figved N, Nyland H (2019) Disability and risk factors for unemployment in chronic fatigue syndrome: a comparison with multiple sclerosis. Fatigue: Biomedicine, Health and Behavior 7 (3): 127.

Pasternak E, Foley F (2018) Comparing relationships between demographic and MS disease factors, objective and subjective fatigue metrics. Multiple Sclerosis Journal Conference: (1 Supplement 1): 84.

Ploughman M, Beaulieu S, Harris C, Hogan S, Manning OJ et al. (2014) The Canadian survey of health, lifestyle and ageing with multiple sclerosis: Methodology and initial results. Bmj open 4 (7):

Ploughman M, Beaulieu S, Harris C, Hogan S, Manning OJ et al. (2014) The Canadian survey of health, lifestyle and ageing with multiple sclerosis: methodology and initial results. Bmj open 4 (7): e005718, 002014.

Ramamurthy G, Ranganathan LN, Kanthimathinathan S, Govindarajan S, Maheswari EU et al. (2018) Cognitive fatigue in multiple sclerosis: Correlation between objective and subjective measures. Neurology Conference: (15 Supplement 1):

Rammohan KW, Li D, Halper J, Murphy SM, Patton L et al. (2019) The economic impact of multiple sclerosis: A preliminary look at the North American registry for care and research in multiple sclerosis (NARCRMS). Multiple Sclerosis Journal Conference: (Supplement 1): 151.

Rasmussen PV, Kobelt G, Berg J, Capsa D, Gannedahl M et al. (2017) New insights into the burden and costs of multiple sclerosis in Europe: Results for Denmark. Multiple Sclerosis 23 (2_suppl): 53.

Salehpoor G, Rezaei S, Hosseininezhad M (2014) Quality of life in multiple sclerosis (MS) and role of fatigue, depression, anxiety, and stress: A bicenter study from north of Iran. Iran J Nurs Midwifery Res 19 (6): 593.

Self MM, Fobian A, Cutitta K, Wallace A, Lotze TE (2018) Health-Related Quality of Life in Pediatric Patients With Demyelinating Diseases: Relevance of Disability, Relapsing Presentation, and Fatigue. J Pediatr Psychol 43 (2): 133.

Simmons RD, Tribe KL, McDonald EA (2010) Living with multiple sclerosis: longitudinal changes in employment and the importance of symptom management. Journal of Neurology 257 (6): 926.

Steinberg J, Cores V, Curbelo MC, Bauer J, Osorio M et al. (2018) Impact of multiple sclerosis on the occupational status in developing countries. An experience in Argentina. Multiple Sclerosis Journal Conference: (2 Supplement): 809.

Strober LB, Christodoulou C, Benedict RH, Westervelt HJ, Melville P et al. (2012) Unemployment in multiple sclerosis: the contribution of personality and disease. Multiple Sclerosis 18 (5): 647.

Tabrizi FM, Radfar M (2015) Fatigue, Sleep Quality, and Disability in Relation to Quality of Life in Multiple Sclerosis. 17 (6): 268.

Tanriverdi D, Okanli A, Sezgin S, Ekinci M (2010) Quality of life in patients with multiple sclerosis in Turkey: relationship to depression and fatigue. Journal of Neuroscience Nursing 42 (5): 267.

Terzi M, Sen S, Kumcagiz H, Terzi Y (2018) Variables that affect life satisfaction of multiple sclerosis patients a comparative study. Multiple Sclerosis Journal Conference: (3): 387.

Teshale SM, Molton IR, Jensen MP (2019) Associations among decisional autonomy, fatigue, pain, and well-being in long-term physical disability. Rehabilitation Psychology 64 (3): 288.

Thomas S, Thomas PW, Kersten P, Jones R, Green C et al. (2013) A pragmatic parallel arm multi-centre randomised controlled trial to assess the effectiveness and cost-effectiveness of a group-based fatigue management programme (FACETS) for people with multiple sclerosis. J Neurol Neurosurg Psychiatry 84 (10): 1092.

Toussaint-Duyster LC, Wong YYM, Van der Cammen-van Zijp MH, Pelt-Gravesteijn DV, Catsman-Berrevoets CE et al. (2018) Fatigue and physical functioning in children with multiple sclerosis and acute disseminated encephalomyelitis. Multiple Sclerosis 24 (7): 982.

Youness H, Abdulhakeem Z, Otmani HE, Elmoutawakkil B, Slassi I et al. (2018) Translation in "Arab" and "Moroccan dialect" adaptation and validation of the modified fatigue impact scale during multiple sclerosis. Multiple Sclerosis Journal Conference: (2 Supplement): 790.

**Study Design**

Coyne KS, Boscoe AN, Currie BM, Landrian AS, Wandstrat TL (2015) Understanding Drivers of Employment Changes in a Multiple Sclerosis Population. International Journal of MS Care 17 (5): 245-252.

Newland PK, Lunsford V, Flach A (2017) The interaction of fatigue, physical activity, and health-related quality of life in adults with multiple sclerosis (MS) and cardiovascular disease (CVD). Applied Nursing Research 33 49.

Popp RFJ, Fierlbeck AK, Knuttel H, Konig N, Rupprecht R et al. (2017) Daytime sleepiness versus fatigue in patients with multiple sclerosis: A systematic review on the Epworth sleepiness scale as an assessment tool. Sleep Medicine Reviews 32 95.

**Outcomes**

Aristotelous P, Stefanakis M, Pantzaris M, Pattichis C, Hadjigeorgiou GM et al. (2019) Associations between functional capacity, isokinetic leg strength, sleep quality and cognitive function in multiple sclerosis patients: a cross-sectional study. Postgraduate Medicine 131 (7): 453.

Barzegar M, Badihian S, Mirmosayyeb O, Ashtari F, Jamadi M et al. (2018) Comparative study of quality of life, anxiety, depression, and fatigue among patients with neuromyelitis optica spectrum disorder and multiple sclerosis: The first report from Iran. Multiple Sclerosis and Related Disorders 22 161.

Baumstarck-Barrau K, Simeoni MC, Reuter F, Klemina I, Aghababian V et al. (2011) Cognitive function and quality of life in multiple sclerosis patients: a cross-sectional study. BMC Neurology 11 17.

Beckmann Y, Ture S, Duman SU (2019) Vitamin D deficiency and its association with fatigue and quality of life in multiple sclerosis patients. EPMA Journal

Berkovich R, Bartolome L, Cambron-Mellott MJ, Bramlett J, Su W et al. (2019) Disability level and comorbidity burden among multiple sclerosis (MS) patients in the US. Neurology Conference: (15 Supplement 1):

Biernacki T, Sandi D, Kincses ZT, Fuvesi J, Rozsa C et al. (2019) Contributing factors to health-related quality of life in multiple sclerosis. Brain Behav 9 (12): e01466, 02019.

Caceres F, Vanotti S, Benedict RH, Group RW (2014) Cognitive and neuropsychiatric disorders among multiple sclerosis patients from Latin America: Results of the RELACCEM study. Multiple Sclerosis and Related Disorders 3 (3): 335.

Colbeck M (2018) Sensory processing, cognitive fatigue, and quality of life in multiple sclerosis: Traitement de l'information sensorielle, fatigue cognitive et qualite de vie des personnes atteintes de sclerose en plaques. Canadian Journal of Occupational Therapy 85 (2): 169.

Concetta I, Magistrale G, Argento O, Pisani V, Battista GD et al. (2015) Occupational stress and personality traits in multiple sclerosis: A preliminary study. Multiple Sclerosis and Related Disorders 4 (4): 315.

Contentti EC, Lopez P, Tkachuk VA, Aguirre MEB, Caride A (2019) Relapse remitting multiple sclerosis and employment in an Argentinean population: Associations with neuropsychological factors. European Journal of Neurology Conference: (Supplement 1): 905.

Covey TJ, Shucard JL, Shucard DW, Stegen S, Benedict RH (2012) Comparison of neuropsychological impairment and vocational outcomes in systemic lupus erythematosus and multiple sclerosis patients. J Int Neuropsychol Soc 18 (3): 530-540.

Culnan E, Tessier J, Grunberg V, Morse C, Germain A et al. (2018) Insomnia symptom severity and quality of life in persons with relapsing-remitting multiple sclerosis. Sleep Conference: (Supplement 1): A383.

Dayapoglu N, Tan M (2011) Quality of life in relapsing-remitting multiple sclerosis. Pakistan Journal of Medical Sciences 27 (1): 133.

Drulovic J, Bursac LO, Milojkovic D, Tepavcevic DK, Gazibara T et al. (2013) MSQoL-54 predicts change in fatigue after inpatient rehabilitation for people with multiple sclerosis. Disability and Rehabilitation 35 (5): 362.

Drulovic J, Mesaros S, Maric G, Martinovic V, Ivanovic J et al. (2019) Working status in persons with multiple sclerosis. Journal of the Neurological Sciences Conference: (WCN 2019). United Arab Emirates. 405 (Supplement): 208.

Effat S, Azzam H, Shalash A, Elkatan S, Elrassas H (2016) Self-reported quality of life of patients with multiple sclerosis with mild disability. Egyptian Journal of Neurology, Psychiatry and Neurosurgery 53 (3): 161.

Eriksson J, Kobelt G, Gannedahl M, Berg J (2019) Association between Disability, Cognition, Fatigue, EQ-5D-3L Domains, and Utilities Estimated with Different Western European Value Sets in Patients with Multiple Sclerosis. Value in Health 22 (2): 231.

Farran N, Safieddine BR, Bayram M, Hanna TA, Massouh J et al. (2020) Factors affecting MS patients' health-related quality of life and measurement challenges in Lebanon and the MENA region. 6 (1): 2055217319848467.

Fernández O, Baumstarck-Barrau K, Simeoni MC, Auquier P (2011) Patient characteristics and determinants of quality of life in an international population with multiple sclerosis: assessment using the MusiQoL and SF-36 questionnaires. Mult Scler 17 (10): 1238-1249.

Fernandez-Munoz JJ, Moron-Verdasco A, Cigaran-Mendez M, Munoz-Hellin E, Perez-de-Heredia-Torres M et al. (2015) Disability, quality of life, personality, cognitive and psychological variables associated with fatigue in patients with multiple sclerosis. Acta Neurologica Scandinavica 132 (2): 118.

Findling O, Baltisberger M, Jung S, Kamm CP, Mattle HP et al. (2015) Variables related to working capability among swiss patients with multiple sclerosis - A cohort study. PLoS One 10 (4):

Findling O, Baltisberger M, Jung S, Kamm CP, Mattle HP et al. (2015) Variables related to working capability among Swiss patients with multiple sclerosis--a cohort study. PLoS One 10 (4): e0121856, 0122015.

Füvesi J, Bencsik K, Losonczi E, Fricska-Nagy Z, Mátyás K et al. (2010) Factors influencing the health-related quality of life in Hungarian multiple sclerosis patients. Journal of the Neurological Sciences 293 (1-2): 59-64.

Ghajarzadeh M, Sahraian MA, Fateh R, Daneshmand A (2012) Fatigue, depression and sleep disturbances in Iranian patients with multiple sclerosis. Acta Med Iran 50 (4): 244.

Glad SB, Nyland H, Aarseth JH, Riise T, Myhr KM (2011) How long can you keep working with benign multiple sclerosis? J Neurol Neurosurg Psychiatry 82 (1): 78.

Gorp DAMv, Hiele Kvd, Heerings MAP, Jongen PJ, Lieshout v et al. (2018) Cognitive functioning as a determinant of employment outcomes in patients with multiple sclerosis; a one-year longitudinal study. Multiple Sclerosis Journal Conference: (2 Supplement): 442.

Gravesande K. Storm van's PC, A. Blaschek KR, P. Huppke LR, V. Mall JK, E. Kalbe VK et al. (2019) The Multiple Sclerosis Inventory of Cognition for Adolescents (MUSICADO): A brief screening instrument to assess cognitive dysfunction, fatigue and loss of health-related quality of life in pediatric-onset multiple sclerosis. European Journal of Paediatric Neurology 23 (6): 792.

Hakansson I, Johansson L, Dahle C, Vrethem M, Ernerudh J (2019) Fatigue scores correlate with other self-assessment data, but not with clinical and biomarker parameters, in CIS and RRMS. Multiple Sclerosis and Related Disorders 36 101424.

Hiele Kvd, Gorp DAMv, Heerings MAP, Jongen PJ, Klink JJLvd et al. (2019) Caregiver strain among life partners of persons with mild disability due to relapsing-remitting multiple sclerosis. Multiple Sclerosis and Related Disorders 31 5.

Hiele Kvd, Gorp Dv, Ruimschotel R, Kamminga N, Visser L et al. (2015) Work Participation and Executive Abilities in Patients with Relapsing-Remitting Multiple Sclerosis. PLoS One 10 (6): e0129228, 0122015.

Hyncicova E, Kalina A, Vyhnalek M, Nikolai T, Martinkovic L et al. (2018) Health-related quality of life, neuropsychiatric symptoms and structural brain changes in clinically isolated syndrome. PLoS One 13 (7): e0200254, 0202018.

Jongen PJ, Lehnick D, Koeman J, Frequin S, Heersema D et al. (2014) Fatigue and health-related quality of life in relapsing-remitting multiple sclerosis after 2 years glatiramer acetate treatment are predicted by changes at 6 months: an observational multi-center study. Journal of Neurology 261 (8): 1469.

Jongen PJ, Lehnick D, Sanders E, Seeldrayers P, Fredrikson S et al. (2010) Health-related quality of life in relapsing remitting multiple sclerosis patients during treatment with glatiramer acetate: a prospective, observational, international, multi-centre study. Health and Quality of Life Outcomes [electronic resource] 8 133.

Karampampa K, Gustavsson A, Miltenburger C, Eckert B (2012) Treatment experience, burden and unmet needs (TRIBUNE) in MS study: results from five European countries. Multiple Sclerosis 18 (2 Suppl): 7.

Kargarfard M, Eetemadifar M, Mehrabi M, Maghzi AH, Hayatbakhsh MR (2012) Fatigue, depression, and health-related quality of life in patients with multiple sclerosis in Isfahan, Iran. European Journal of Neurology 19 (3): 431.

Ketelslegers IA, Catsman-Berrevoets CE, Boon M, Eikelenboom MJ, Stroink H et al. (2010) Fatigue and depression in children with multiple sclerosis and monophasic variants. Europ J Paediatr Neurol 14 (4): 320.

Klevan G, Jacobsen CO, Aarseth JH, Myhr KM, Nyland H et al. (2014) Health related quality of life in patients recently diagnosed with multiple sclerosis. Acta Neurologica Scandinavica 129 (1): 21.

Kratz AL, Braley TJ, Foxen-Craft E, Scott E, Murphy JF et al. (2017) How Do Pain, Fatigue, Depressive, and Cognitive Symptoms Relate to Well-Being and Social and Physical Functioning in the Daily Lives of Individuals With Multiple Sclerosis? Archives of Physical Medicine and Rehabilitation 98 (11): 2160.

Krause I, Kern S, Horntrich A, Ziemssen T (2013) Employment status in multiple sclerosis: impact of disease-specific and non-disease-specific factors. Multiple Sclerosis 19 (13): 1792.

Kurnaz AHM, Unlu MD, Demirci S (2019) Prevalence of comorbidities in relapsing-remitting and secondary progressive multiple sclerosis. Journal of the Neurological Sciences Conference: (WCN 2019). United Arab Emirates. 405 (Supplement): 199.

L. Barin AS, G. Disanto HB, P. Calabrese AC, C.P. Kamm JK, J. Kuhle CG et al. (2018) The disease burden of Multiple Sclerosis from the individual and population perspective: Which symptoms matter most? Multiple Sclerosis and Related Disorders 25 112.

L.I. Berrigan JDF, S.B. Patten HT, C. Wolfson SW, K.M. Fiest KAM, R.A. Marrie CTitE (2016) Health-related quality of life in multiple sclerosis: Direct and indirect effects of comorbidity. Neurology 86 (15): 1417.

Maurer M, Comi G, Freedman MS, Kappos L, Olsson TP et al. (2016) Multiple sclerosis relapses are associated with increased fatigue and reduced health-related quality of life - A post hoc analysis of the TEMSO and TOWER studies. Multiple Sclerosis and Related Disorders 7 33.

McBurney RN, Chen M, Schmidt H, Loud S, Kolaczkowski L (2018) Differences in symptoms, functioning and quality of life between people with relapsing versus progressive forms of multiple sclerosis. Multiple Sclerosis Journal Conference: (1 Supplement 1): 107.

Messinis L, Kosmidis MH, Nasios G, Konitsiotis S, Ntoskou A et al. (2020) Do Secondary Progressive Multiple Sclerosis patients benefit from Computer- based cognitive neurorehabilitation? A randomized sham controlled trial. Multiple Sclerosis and Related Disorders 39 101932.

Mikaeloff Y, Rollot F, Tremolieres L, Leray E, Casey R et al. (2018) Juvenile multiple sclerosis (SOKIDMUS study): Evaluation of factors associated with socio-professional performances in adulthood. Multiple Sclerosis Journal Conference: (2 Supplement): 134.

Mikula P, Nagyova I, Krokavcova M, Vitkova M, Rosenberger J et al. (2015) The mediating effect of coping on the association between fatigue and quality of life in patients with multiple sclerosis. Psychology, Health & Medicine 20 (6): 653.

Motl RW, Suh Y, Weikert M (2010) Symptom cluster and quality of life in multiple sclerosis. Journal of Pain and Symptom Management 39 (6): 1025.

Muto M, Mori M, Sato Y, Uzawa A, Masuda S et al. (2015) Current symptomatology in multiple sclerosis and neuromyelitis optica. European Journal of Neurology 22 (2): 299.

Nazareth TA, Rava AR, Polyakov JL, Banfe EN, Ii RWW et al. (2018) Relapse prevalence, symptoms, and health care engagement: patient insights from the Multiple Sclerosis in America 2017 survey. Multiple Sclerosis and Related Disorders 26 219.

Newland PK, Flick LH, Thomas FP, Shannon WD (2014) Identifying symptom co-occurrence in persons with multiple sclerosis. 23 (5): 529.

Nicholas JA, Electricwala B, Lee LK, Johnson KM (2019) Burden of relapsing-remitting multiple sclerosis on workers in the US: a cross-sectional analysis of survey data. BMC Neurology 19 (1): 258.

Nourbakhsh B, Julian L, Waubant E (2016) Fatigue and depression predict quality of life in patients with early multiple sclerosis: a longitudinal study. European Journal of Neurology 23 (9): 1482.

Ostojic S, Stevanovic D, Jancic J (2016) Quality of life and its correlates in adolescent multiple sclerosis patients. Multiple Sclerosis and Related Disorders 10 57.

Papuc E, Stelmasiak Z (2012) Factors predicting quality of life in a group of Polish subjects with multiple sclerosis: accounting for functional state, socio-demographic and clinical factors. Clinical Neurology and Neurosurgery 114 (4): 341.

Patti F, Vila C (2014) Symptoms, prevalence and impact of multiple sclerosis in younger patients: a multinational survey. Neuroepidemiology 42 (4): 211.

Planche V, Moisset X, Morello R, Dumont E, Gibelin M et al. (2017) Improvement of quality of life and its relationship with neuropsychiatric outcomes in patients with multiple sclerosis starting treatment with natalizumab: A 3-year follow-up multicentric study. Journal of the Neurological Sciences 382 148.

Purmonen T, Hakkarainen T, Tervomaa M, Ruutiainen J (2019) Impact of multiple sclerosis phenotypes on burden of disease in Finland. Journal of Medical Economics 1.

Reese JP, Wienemann G, John A, Linnemann A, Balzer-Geldsetzer M et al. (2013) Preference-based Health status in a German outpatient cohort with multiple sclerosis. Health and Quality of Life Outcomes [electronic resource] 11 162.

Renner A, Baetge SJ, Filser M, Stute N, Penner IK (2018) Employment status in individuals with multiple sclerosis: Which predictors are important in different disease subtypes? Multiple Sclerosis Journal Conference: (2 Supplement): 653.

Roessler RT, Rumrill Jr PD, Li J, Leslie MJ (2015) Predictors of differential employment statuses of adults with multiple sclerosis. Journal of Vocational Rehabilitation 42 141-152.

Rommer PS, Eichstadt K, Ellenberger D, Flachenecker P, Friede T et al. (2019) Symptomatology and symptomatic treatment in multiple sclerosis: Results from a nationwide MS registry. Multiple Sclerosis 25 (12): 1641.

Rooney S, McFadyen DA, Wood DL, Moffat DF, Paul PL (2019) Minimally important difference of the fatigue severity scale and modified fatigue impact scale in people with multiple sclerosis. Multiple Sclerosis and Related Disorders 35 158.

Roppolo M, Mulasso A, Gollin M, Bertolotto A, Ciairano S (2013) The role of fatigue in the associations between exercise and psychological health in Multiple Sclerosis: Direct and indirect effects. Mental Health and Physical Activity 6 (2): 87.

Rosato R, Testa S, Oggero A, Molinengo G, Bertolotto A (2015) Quality of life and patient preferences: identification of subgroups of multiple sclerosis patients. Quality of Life Research 24 (9): 2173.

Ross AP, Williamson A, Smrtka J, Tracy TF, Saunders C et al. (2013) Assessing relapse in multiple sclerosis questionnaire: results of a pilot study. Mult Scler Int 2013 470476.

Rosti-Otajarvi E, Hamalainen P, Wiksten A, Hakkarainen T, Ruutiainen J (2017) Validity and reliability of the Fatigue Severity Scale in Finnish multiple sclerosis patients. Brain Behav 7 (7): e00743, 02017.

Sandvig I, Barlinn J, Nedregaard B, Skjeldal OH (2015) Multiple sclerosis in children and adolescents. An important differential diagnosis of acute neurological disease. Europ J Paediatr Neurol 19 (2): 211.

Smedal T, Beiske AG, Glad SB, Myhr KM, Aarseth JH et al. (2011) Fatigue in multiple sclerosis: associations with health-related quality of life and physical performance. European Journal of Neurology 18 (1): 114.

Smedema SM (2020) An analysis of the relationship of character strengths and quality of life in persons with multiple sclerosis. Quality of Life Research 2020 Jan 03

Sterz C ED, Meißner H, Friede T, Flachenecker P. (2016) Employment-associated factors in multiple sclerosis: Results of a cross-sectional study in Germany. Edorium J Disabil Rehabil 2 24-33.

Strober LB (2018) Quality of life and psychological well-being in the early stages of multiple sclerosis (MS): Importance of adopting a biopsychosocial model. Disabil Health J 11 (4): 555.

Strober LB, Arnett PA (2016) Unemployment among women with multiple sclerosis: the role of coping and perceived stress and support in the workplace. Psychol Health Med 21 (4): 496-504.

Szilasiova J, Krokavcova M, Gdovinova Z, Rosenberger J, Dijk JPv (2011) Quality of life in patients with multiple sclerosis in Eastern Slovakia. Disability and Rehabilitation 33 (17-18): 1587.

Tecchio F, Cancelli A, Cottone C, Zito G, Pasqualetti P et al. (2014) Multiple sclerosis fatigue relief by bilateral somatosensory cortex neuromodulation. Journal of Neurology 261 (8): 1552.

Tepavcevic DK, Pekmezovic T, Stojsavljevic N, Kostic J, Basuroski ID et al. (2014) Change in quality of life and predictors of change among patients with multiple sclerosis: a prospective cohort study. Quality of Life Research 23 (3): 1027.

Vanotti S, Ciufia N, Eizaguirre M, Cabral N, Yastremiz C et al. (2019) Cognitive and clinical predictors of employment status among patients with multiple sclerosis in Argentina. Neurology Conference: (15 Supplement 1):

Vanotti S, Eizaguirre M, Cabral N, Yastremiz C, Silva B et al. (2018) Employment status of people with multiple sclerosis in Argentina. Multiple Sclerosis Journal Conference: (2 Supplement): 241.

Viana P, Rodrigues E, Fernandes C, Matas A, Barreto R et al. (2015) InMS: Chronic insomnia disorder in multiple sclerosis - a Portuguese multicentre study on prevalence, subtypes, associated factors and impact on quality of life. Multiple Sclerosis and Related Disorders 4 (5): 477.

Williams AE, Vietri JT, Isherwood G, Flor A (2014) Symptoms and Association with Health Outcomes in Relapsing-Remitting Multiple Sclerosis: Results of a US Patient Survey. Mult Scler Int 2014 203183.

Wunderlich M, Heesen C, Haase R, Stellmann JP, Angstwurm K et al. (2018) Early stages of disability in patients with multiple sclerosis by physician and patient-reported outcomes: A two-year study. Multiple Sclerosis Journal Conference: (2 Supplement): 923.

Yalachkov Y, Soydas D, Bergmann J, Frisch S, Behrens M et al. (2019) Determinants of quality of life in relapsing-remitting and progressive multiple sclerosis. Multiple Sclerosis and Related Disorders 30 33.

Yamout B, Issa Z, Herlopian A, Bejjani ME, Khalifa A et al. (2013) Predictors of quality of life among multiple sclerosis patients: a comprehensive analysis. European Journal of Neurology 20 (5): 756.

Zafar AB, Ness J, Dowdy S, Avis K, Bashir K (2012) Examining sleep, fatigue, and daytime sleepiness in pediatric multiple sclerosis patients. Multiple Sclerosis 18 (4): 481.

Zarei S, Maldonado I, Franqui-Dominguez L, Rubi C, Rosa YT et al. (2019) Impact of delayed treatment on exacerbations of multiple sclerosis among Puerto Rican patients. Surg Neurol Int 10:200, 2019.

Ziemssen T, Tolley C, Bennett B, Kilgariff S, Jones E et al. (2019) A mixed methods approach towards understanding key disease characteristics associated with the progression from RRMS to SPMS: Physicians' and patients' views. Multiple Sclerosis and Related Disorders 38 101861.

Ziemssen T, Tolley C, Bennett B, Kilgariff S, Jones E et al. (2020) A mixed methods approach towards understanding key disease characteristics associated with the progression from RRMS to SPMS: Physicians' and patients' views. Multiple Sclerosis and Related Disorders 38 101861.

**On-topic SLR/MA/NMA**

Carroll S, Chalder T, Hemingway C, Heyman I, Moss-Morris R (2016) Understanding fatigue in paediatric multiple sclerosis: A systematic review of clinical and psychosocial factors. Developmental Medicine and Child Neurology 58 (3): 229.

Gerhard L, Dorstyn DS, Murphy G, Roberts RM (2018) Neurological, physical and sociodemographic correlates of employment in multiple sclerosis: A meta-analysis. Journal of Health Psychology 25 (1): 92-104.

Kheradmand M, Afshari M, Nasehi MM, Aghaei I, Shabani M et al. (2019) Prevalence of subtypes of multiple sclerosis and the most common clinical symptoms in Iranian patients: A meta-analysis. Clinical and Experimental Neuroimmunology 10 (1): 33.

Krupp LB, Goble JA, Toledano H, Thompson M, Pervaiz N et al. (2018) Systematic review of the burden of pediatric multiple sclerosis in north america and europe. Value in Health Conference: (Supplement 3): S350.

Raggi A, Covelli V, Schiavolin S, Scaratti C, Leonardi M et al. (2016) Work-related problems in multiple sclerosis: a literature review on its associates and determinants. Disability and Rehabilitation 38 (10): 936.

Schiavolin S, Leonardi M, Giovannetti AM, Antozzi C, Brambilla L et al. (2013) Factors related to difficulties with employment in patients with multiple sclerosis: a review of 2002-2011 literature. International Journal of Rehabilitation Research 36 (2): 105.

**Duplicate**

Chen J, Taylor B, Palmer AJ, Kirk-Brown A, Dijk Pv et al. (2019) Estimating MS-related work productivity loss and factors associated with work productivity loss in a representative Australian sample of people with multiple sclerosis. Multiple Sclerosis Journal 25 (7): 994.

Florea A, Maurey H, Sauter ML, Bellesme C, Sevin C et al. (2020) Fatigue, depression, and quality of life in children with multiple sclerosis: a comparative study with other demyelinating diseases. Developmental Medicine and Child Neurology 62 (2): 241.

Gorp DAMv, Hiele Kvd, Heerings MAP, Jongen PJ, Klink JJLvd et al. (2019) Cognitive functioning as a predictor of employment status in relapsing-remitting multiple sclerosis: a 2-year longitudinal study. Neurological Sciences 40 (12): 2555.

Gravesande KSvs, Blaschek A, Calabrese P, Rostasy K, Huppke P et al. (2019) Fatigue and depression predict health-related quality of life in patients with pediatric-onset multiple sclerosis. Multiple Sclerosis and Related Disorders 36 101368.

Razazian N, Shokrian N, Bostani A, Moradian N, Tahmasebi S (2014) Study of fatigue frequency and its association with sociodemographic and clinical variables in patients with multiple sclerosis. Neurosciences 19 (1): 38.

Weiland TJ, Livera AMD, Brown CR, Jelinek GA, Aitken Z et al. (2019) Health outcomes and lifestyle in a sample of people with multiple sclerosis (HOLISM): Longitudinal and validation cohorts. Multiple Sclerosis Journal Conference: (3): 445.

**Unavailable**

Fox RJ, Bacon TE, Chamot E, Salter AR, Cutter GR et al. (2015) Prevalence of multiple sclerosis symptoms across lifespan: data from the NARCOMS Registry. Neurodegener Dis Manag 5 (6 Suppl): 3.
