# Supplementary material for: Prevalence and burden of multiple sclerosis-related fatigue: a systematic literature review
Source: BMC Neurol. 2021 Dec 2;21:468. doi: 10.1186/s12883-021-02396-1 (PMC8638268; doi:10.1186/s12883-021-02396-1)
Supplement: Supplementary file 3 — Additional file 3: Study and baseline characteristics of included studies. File includes three tables: 1) Study and baseline characteristics –Epidemiology; 2) Study and baseline characteristics – Economic burden (fatigue assessed as categorical); and 3) Study and baseline characteristics – Humanistic burden. [file 12883_2021_2396_MOESM3_ESM.docx]

**Title:** Prevalence and burden of multiple sclerosis-related fatigue: a systematic literature review

**Authors:** Abril Oliva Ramirez, MEpi^1^; Alexander Keenan, MA, MPH^2^; Olivia Kalau^1^; Evelyn Worthington, MSc^1^; Lucas Cohen, MSc^1^; Sumeet Singh, MSc, RPh^1^

^1^EVERSANA, Burlington, Ontario, Canada

^2^Health Economics and Market Access, Janssen Research & Development, LLC, Titusville, NJ, USA

Corresponding author: Alexander Keenan, [AKeenan1@its.jnj.com](mailto:AKeenan1@its.jnj.com), Janssen Scientific Affairs Titusville, NJ, USA

## **Study and baseline characteristics of included studies**

Supplementary Table 1: Study and baseline characteristics - Epidemiology

| Author (year) | Country | Sample Size | Study Design | Mean Age (years) | Female (%) | RRMS (%) | Mean Disease Duration (years) | Mean EDSS | Outcome(s) |
| --- | --- | --- | --- | --- | --- | --- | --- | --- | --- |
| **Adult** | | | | | | | | | |
| Alvarenga-Filho (2015) | Brazil | 31 | Cross-sectional | NR | NR | 100 | NR | NR | Prevalence |
| Anens (2014) | Sweden | 287 | Cross-sectional | 51.5 | 70.7 | 47.0 | NR | NR | Prevalence |
| Battaglia (2017) | Italy | 1010 | Cross-sectional/Retrospective | 45.0 | 70.4 | 66.7 | NR | 3.7 | Prevalence |
| Calabrese (2017) | Switzerland | 721 | Cross-sectional/Retrospective | 48.4 | 74.3 | 60.6 | NR | 3.1 | Prevalence |
| Fiest (2016) | Canada | 949 | Longitudinal | 48.6 | 75.2 | 72.4 | 15.4 | NR | Prevalence, Incidence |
| Flachenecker (2017) | Germany | 5475 | Cross-sectional/Retrospective | 51.8 | 74.3 | 45.6 | NR | 4.0 | Prevalence |
| Fricska-Nagy (2016) | Hungary | 428 | Cross-sectional | 43.6 | 73.7 | 100 | 11.2 | NR | Prevalence |
| Hadgkiss (2013) | International | 2239 | Cross-sectional | NR | 82.2 | 76.6 | NR | NR | Prevalence |
| Havrdova (2017) | Czech Republic | 747 | Cross-sectional/Retrospective | 46.7 | 74.8 | 58.1 | NR | 3.5 | Prevalence |
| Kratz (2016) | USA | 180 | Cross-sectional | 50.5 | 78.0 | 56.0 | 13.0 | NR | Prevalence |
| Labuz-Roszak (2012) | Poland | 122 | Cross-sectional | 37.7 | 71.3 | 75.4 | 6.7 | 2.2 | Prevalence |
| Larnaout (2018) ^a^ | NR | 70 | Cross-sectional cut of longitudinal | 36.0 | NR | NR | NR | 2.0 | Prevalence |
| Lebrun-Frenay (2017) | France | 491 | Cross-sectional/Retrospective | 47.2 | 74.3 | 61.3 | NR | 3.6 | Prevalence |
| Oreja-Guevara (2017) | Spain | 462 | Cross-sectional/Retrospective | 42.6 | 66.7 | 72.5 | NR | 3.4 | Prevalence |
| Pentek (2017) | Hungary | 521 | Cross-sectional/Retrospective | 46.9 | 78.3 | 55.3 | NR | 3.9 | Prevalence |
| Pokryszko-Dragan (2016) | Poland | 44 | Cross-sectional | 31.4 | 61.4 | CIS | 0.0 ^d^ | 1.4 | Prevalence |
| Reilly (2017) | International ^b^ | 2518 | Cross-sectional | 45.7 | 82.7 | 61.8 | NR | NR | Prevalence |
| Rooney (2019) | International ^c^ | 412 | Cross-sectional | 46.0 | 81.3 | 70.6 | 9.6 | NR | Prevalence |
| Runia (2015) | Netherlands | 127 | Cross-sectional cut of longitudinal | 34.0 | 77.2 | CIS | NR | NR | Prevalence |
| Selmaj (2017) | Poland | 411 | Cross-sectional/Retrospective | 39.7 | 73.5 | 63.7 | NR | 3.5 | Prevalence |
| Thompson (2017) | UK | 779 | Cross-sectional/Retrospective | 56.7 | 70.1 | 36.7 | NR | 5.5 | Prevalence |
| Uitdehaag (2017) | Netherlands | 382 | Cross-sectional/Retrospective | 54.0 | 71.7 | 35.1 | NR | 4.9 | Prevalence |
| van der Vuurst de Vries (2017) | Netherlands | 235 | Cross-sectional cut of longitudinal | 34.2 ^d^ | 75.7 | CIS | 0.25 | NR | Prevalence |
| von Bismarck (2018) | Germany | 1124 | Cross-sectional cut of longitudinal | NR | 69.0 | 55.3 | NR | NR | Prevalence |
| Weiland (2015) | International | 2138 | Cross-sectional | 45.5 | 82.3 | 61.5 | 8.5 | NR | Prevalence |
| Weiland (2019) ^e^ | International | **1401**  1401  *573* | Longitudinal (HOLISM); cross-sectional (validation) | **45.9**  48.4  *46.2* | **82.7**  82.7  *80.8* | **63.3**  59.2  *67.4* | **NR**  NR  *NR* | **NR**  NR  *NR* | Prevalence |
| Wood (2013) | Australia | 198 | Longitudinal | 48.2 | 69.2 | 74.7 | NR | NR | Prevalence |
| **Pediatric** | | | | | | | | | |
| Florea (2019) | France | 26 | Cross-sectional cut of longitudinal | 15.2 | 65.4 | Pediatric | 2.8 | 0.1 | Prevalence |
| Goretti (2010) | Italy | 56 | Cross-sectional | 17.2 | 50 | 100 | 5.5 | 1.7 | Prevalence |
| Parrish (2013) | USA | 36 | Cross-sectional | 14.1 | 69.4 | Pediatric | 2.1 | 1.0 | Prevalence |
| van’s Gravesande (2019) | Germany and Austria | 106 | Cross-sectional | 15.7 | 71.7 | 100 | 1.55 | 0.7 | Prevalence |
| **Mixed or unknown age** | | | | | | | | | |
| Garcia (2019) ^a, e^ | Brazil | **38**  26 | Longitudinal | **NR**  NR | **NR**  NR | **100**  100 | **NR**  NR | **NR**  NR | Prevalence |
| Kaya Aygunoglu (2015) | Turkey | 120 | Cross-sectional | 34.2 | 70.0 | 84.2 | 8.1 | 2.9 | Prevalence |
| Razazian (2014) | Iran | 300 | Cross-sectional | 35.3 | 72.0 | 83.4 | 7.3 | NR | Prevalence |
| Rupprecht (2018) ^a^ | Germany | 2052 | Cross-sectional | NR | 70.0 | 76.9 | NR | NR | Prevalence |

^a^ Conference abstract.

^b^ Australasia (Australia and New Zealand), Europe, North America (United States and Canada).

^c^ UK, USA, and Australia (countries where organizations shared the open-access survey).

^d^ Age at time of CIS. Age at baseline was not reported; however, patients were included in the study within 6 months after the onset of CIS.

^e^ Baseline data are presented in **bold** text.

Abbreviations: CIS = clinically isolated syndrome; EDSS = Expanded Disability Status Scale; NR = not reported, RRMS = relapsing-remitting multiple sclerosis.

Supplementary Table 2: Study and baseline characteristics – Economic burden (fatigue assessed as categorical)

| Author (year) | Country | Sample Size | Study Design | Mean Age (years) | Female (%) | RRMS (%) | Mean Disease Duration (years) | Mean EDSS | Outcome(s) |
| --- | --- | --- | --- | --- | --- | --- | --- | --- | --- |
| **Direct costs** | | | | | | | | | |
| da Silva (2016) | Brazil | 210 | Cross-sectional | 40.7 | 70.0 | 79.0 | 7.9 | NR | Costing |
| McKay (2018) | Canada | 340 | Longitudinal | 48.4 | 73.8 | NR ^a^ | 15.1 | NR | Resource utilization |
| **Indirect costs** | | | | | | | | | |
| Doesburg (2019) | Netherlands | 90 | Cross-sectional | 39.3 | 73.3 | 88.9 | NR | NR | Employment status |
| Grytten (2017) | Norway | 93 | Longitudinal | 39.9 | 68.8 | 90.0 | NR | 3.3 | Employment status |
| Koziarska (2018) | Poland | 150 | Cross-sectional | 40.6 | 65.3 | 87.0 | 4.5 | 2.4 | Employment status |
| Lorefice (2018) | Italy | 123 | Cross-sectional | 37.9 | 69.1 | NR ^b^ | 9.6 | 2.4 | Employment status |
| Razazian (2014) | Iran | 300 | Cross-sectional | 35.3 | 72.0 | 83.4 | 7.3 | NR | Employment status |
| Salter (2017) | USA and Canada | 5173 | Cross-sectional | 54.4 | 81.7 | NR | NR | NR | Employment status |
| Weiland (2015) | International | 2138 | Cross-sectional | 45.5 | 81.5 | 61.2 | 8.5 | NR | Employment status |

^a^ 94.4% relapsing MS, not defined if that includes progressive relapsing MS (i.e., secondary progressive MS).

^b^ 83.7% relapsing MS, not defined if that includes progressive relapsing MS (i.e., secondary progressive MS).

Abbreviations: EDSS = Expanded Disability Status Scale; NR = not reported; RRMS = relapsing-remitting multiple sclerosis.

Supplementary Table 3: Study and baseline characteristics – Humanistic burden

| Author (year) | Country | Sample Size | Study Design | Mean Age (years) | Female (%) | RRMS (%) | Mean Disease Duration (years) | Mean EDSS | Outcome(s) Reported |
| --- | --- | --- | --- | --- | --- | --- | --- | --- | --- |
| Cioncoloni (2014) | Italy | 57 | Longitudinal | 41.7 | 68.4 | 80.7 | 9.1 | NR | SF-36 |
| Filho (2019) ^a^ | Brazil | 31 | Cross-sectional | NR | NR | 100 | NR | NR | SF-36 |
| Fricska-Nagy (2016) | Hungary | 428 | Cross-sectional | 43.6 | 73.7 | 100 | 11.2 | NR | MSQOL-54 |
| Goksel Karatepe (2011) | Turkey | 79 | Cross-sectional | 40.6 | 69.6 | 73.4 | NR | 3.2 | MSQOL-54 |
| Gullo (2019) | Australia | 74 | Cross-sectional | 52.7 | 79.7 | 66.2 | NR | NR | SF-36 |
| Kaya Aygunoglu (2015) | Turkey | 120 | Cross-sectional | 34.2 | 70.0 | 84.2 | 8.1 | 2.9 | MSQOL-54 |
| Leonavicius (2016) | Lithuania | 137 | Cross-sectional | 44.7 | 72.3 | 100 | 12.6 | 3.8 | SF-36 |
| Schmidt (2019) | Germany | 260 | Cross-sectional/Retrospective | 44.5 | 79.6 | 66.9 | 10.6 | 3.3 | MusiQoL |
| Takemoto (2015) | Brazil | 210 | Cross-sectional | 40.7 | 70.0 | 79.0 | 7.9 | NR | EQ-5D (utilities) |
| Taveira (2019) | Brazil | 39 | Cross-sectional | 39.1 | 84.6 | 100 | 7.8 ^b^ | 2.3 | FAMS |
| Weiland (2015) | International | 2138 | Cross-sectional | 45.5 | 81.5 | 61.2 | 8.5 | NR | MSQOL-54 |

^a^ Conference abstract.

^b^ Converted from months.

Abbreviations: EDSS = Expanded Disability Status Scale; EQ-5D = EuroQoL-5D; FAMS = Functional Assessment of Multiple Sclerosis; FIS = Fatigue Impact Scale; FSMC = Fatigue Scale for Motor and Cognitive functions; FSS = Fatigue Severity Score; MSQOL-54 = Multiple Sclerosis Quality of Life-54; MusiQoL = Multiple Sclerosis International Quality of Life questionnaire; NR = not reported; RRMS = relapsing-remitting multiple sclerosis; SF-36 = 36-item Short Form health survey.
