# Supplementary material for: Prevalence and burden of multiple sclerosis-related fatigue: a systematic literature review
Source: BMC Neurol. 2021 Dec 2;21:468. doi: 10.1186/s12883-021-02396-1 (PMC8638268; doi:10.1186/s12883-021-02396-1)
Supplement: Supplementary file 4 — Additional file 4: Economic studies reporting fatigue as a linear variable. File includes two tables: 1) Baseline and study characteristics for economic studies reporting fatigue as a linear variable; and 2) Results of economic studies reporting fatigue as a linear variable. [file 12883_2021_2396_MOESM4_ESM.docx]

**Title:** Prevalence and burden of multiple sclerosis-related fatigue: a systematic literature review

**Authors:** Abril Oliva Ramirez, MEpi^1^; Alexander Keenan, MA, MPH^2^; Olivia Kalau^1^; Evelyn Worthington, MSc^1^; Lucas Cohen, MSc^1^; Sumeet Singh, MSc, RPh^1^

^1^EVERSANA, Burlington, Ontario, Canada

^2^Health Economics and Market Access, Janssen Research & Development, LLC, Titusville, NJ, USA

Corresponding author: Alexander Keenan, [AKeenan1@its.jnj.com](mailto:AKeenan1@its.jnj.com), Janssen Scientific Affairs Titusville, NJ, USA

## **Economic studies reporting fatigue as a linear variable**

Supplementary Table 4: Baseline and study characteristics for economic studies reporting fatigue as a linear variable

| **Author (year)** | **Country** | **Sample Size** | **Study Design** | **Mean Age (years)** | **Female (%)** | **RRMS (%)** | **Mean Disease Durations (years)** | **Mean EDSS** | **Outcome** |
| --- | --- | --- | --- | --- | --- | --- | --- | --- | --- |
| Beier (2019) | USA | 407 | Cross-sectional cut of longitudinal study | 53.0 | 83.0 | 57.0 | 14.6 | NR | Employment status |
| Boe Lunde (2014) | Norway | 213 | Cross-sectional | 32.7 | 69.0 | 52.6 | 18.9 | NR | Employment status |
| Cadden (2015) | USA | 53 | Cross-sectional cut of longitudinal study | 51.7 | 85.0 | 57.0 | 15.0 | 4.2 | Employment status |
| Carnero Contentti (2018)* | Argentina | 171 | Cross-sectional | NR | NR | 100 | NR | NR | Employment status |
| Chen (2019) | Australia | 2, 211 | Cross-sectional | NR^†^ | 81.4 | 73.5 | NR^†^ | NR^‡^ | Employment status, Work productivity |
| Doesburg (2018)* | NR | 92 | Cross-sectional | 39.3 | NR | 88.0 | 0.7 | NR | Sick Leave |
| Flensner (2013) | Sweden | 257 | Cross-sectional | 47.5 | 76.3 | 72.8 | NR | NR | Capacity to work |
| Forslin (2018) | Sweden | 116 | Longitudinal | 41.0 | 67.0 | 73.0 | 11.0 | NR | Employment status |
| Glanz (2012) | USA | 337 | Cross-sectional cut of a longitudinal study | 45.4 | 76.0 | 94.7 | 12.4 | NR | Absenteeism, Presenteeism, Overall work impairment, Activity impairment |
| Jongen (2014) | Netherlands | 33 | Cross-sectional cut of a longitudinal study | 39.8 | 75.8 | 57.6 | 1.1 | 1.3 | Working hours |
| Moore (2013) | United Kingdom | 221 | Cross-sectional | 46.0 | 72.4 | 56.1 | 12.3 | 3.0 | Employment status |
| Ness (2018)* | Germany | 559 | Cross-sectional cut of longitudinal study | NR | NR | 100 | NR | NR | Occupational disability |
| Povolo (2019) | Canada | 158 | Cross-sectional/Retrospective | 43.1 | 65.2 | 81.0 | 7.8 | NR^‡^ | Employment status |
| Reese (2011) | Germany | 144 | Cross-sectional | 41.7 | 68.8 | 67.0 | 7.1 | 3.5 | Economic burden |
| Strober (2018) | USA | 40 | Cross-sectional cut of longitudinal study | NR^†^ | 95.0 | 100 | NR^†^ | NR | Employment status |
| Van der Hiele (2014) | Netherlands | 44 | Cross-sectional | NR^†^ | 88.6 | 100 | NR^†^ | NR | Employment status |
| Van der Hiele (2018)* | NR | 161 | Cross-sectional cut of longitudinal study | NR^§^ | 76.0 | 100 | NR | NR^§^ | Employment status, Presenteeism, Work ability, Work difficulties |
| van Gorp (2018) | Netherlands | 163 | Cross-sectional | 42.5 | 77.3 | 100 | NR^§^ | NR^§^ | Employment capability set |
| van Gorp (2019) | Netherlands | 124 | Longitudinal | 42.3 | 82.3 | 100 | 7.6 | 2.1 | Employment status |
| Wickström (2014) | Sweden | 115 | Longitudinal | 39.4 | 73.9 | 100 | 8.8 | 3.0 | Sickness benefits |

*Conference abstracts

† Value not reported for overall cohort but is reported by respective subgroups

‡ Median value reported by respective subgroups

§ Median value reported overall

Abbreviations: NR = Not reported, EDSS = Expanded Disability Status Scale, RRMS = relapsing-remitting multiple sclerosis.

Supplementary Table 5: Results for economic studies reporting fatigue as a linear variable

| **Author (year)** | **Type of analysis** | **Sample size** | **Outcome** | **Predictor(s)** | **Value** | **95% CI** | **p-value** |
| --- | --- | --- | --- | --- | --- | --- | --- |
| Beier (2019) | Multivariate logistic regression | 407 | Unemployment | Age, education, EDSS, MS symptoms duration, MS type, PHQ-9, PSS, **PROMIS fatigue**, Neuro-QoL (general concerns and executive functioning) | OR = 0.98 | 0.94, 1.03 | >0.05 |
| Boe Lunde (2014) | Multivariate logistic regression | 213 | Employment | Age at onset, disease duration, education, EDSS, **FSS** | OR = 0.73 | 0.57, 0.93 | 0.011 |
| Cadden (2015) | Multivariate logistic regression | 53 | Employment status | Cognitive, motor, fatigue, and depression composite scores | OR = 2.73 | 0.77, 9.70 | 0.12 |
| Carnero Contentti (2018)* † | Logistic regression | 171 | Unemployment | NR; included **FSS** | NR | NR | 0.01 |
| Chen (2019) | Log-binomial regression | 1471 | Not being in the labor force | **FSS** | PR = 1.19 | 1.15, 1.23 | <0.001 |
|  | Cragg hurdle model | 740 | Work productivity loss |  | Regression coefficient = 7.40 | 5.78, 9.03 | <0.001 |
| Doesburg (2018)* † | Backward regression | 81 | High sick leave | NR; includes **NFI-MS** | OR = 15.6 | NR | NR^‡^ |
| Flensner (2013) | Multiple logistic regression | 256 | Capacity for work | **FSS-mean**, heat sensitivity, PDQ, HADS, EDSS, age, sex, level of education | OR = 0.75 | 0.57, 0.99^§^ | 0.041 |
| Forslin (2018) | Multivariate logistic regression | 116 | Full- /part-time vs no work | Age, education level, MSIS-physical, **FSS,** NHPT, full-time work, part-time work, Frenchay Activities Index | OR = 2.03 | 1.08, 3.84 | 0.03 |
|  |  |  | Full-time vs part-time or no work |  | OR = 0.65 | 0.38, 1.10 | 0.11 |
| Glanz (2012) | Multivariate logistic regression | 377 | Absenteeism (WPAI:GH) | EDSS, disease duration, CES-D, **MFIS**, STAI-Trait, SDMT | OR = 1.05^§^ | 0.89, 1.25^§^ | NR |
|  |  |  | Presenteeism (WPAI:GH) |  | OR = 1.51^§^ | 1.13, 1.79^§^ | NR |
|  |  |  | Overall work impairment (WPAI:GH) |  | OR = 1.62^§^ | 1.22, 2.01^§^ | NR |
|  |  |  | Activity impairment (WPAI:GH) |  | OR = 2.41^§^ | 2.03, 2.86^§^ | NR |
| Jongen (2014) ^¶^ | Stepwise regression analysis | 33 | Max. hours worked per day | MFIS-5, episodic memory score, working memory score | R^2^ = 0.271 | NA | NR |
|  |  |  | Hours worked per week | MFIS-5, episodic Memory | R^2^ = 0.370 | NA | NR |
| Moore (2013) | Multinomial logistic regression | 221 | No change to employment vs left employment | EDSS, years of education, disease duration, **fatigue (five-point Likert scale)** | OR = 0.66 | 0.40, 1.10 | 0.112 |
|  |  |  | Changes to employment vs left employment |  | OR = 1.15 | 0.71, 1.84 | 0.575 |
|  |  |  | Changes to employment vs no change to employment |  | OR = 1.73 | 1.12, 2.67 | 0.013 |
| Ness (2018)* † | Logistic regression | 559 | Occupational disability | NR; includes **UK-NDS fatigue** | OR = 1.2 | NR | <0.05 |
| Povolo (2019) | SEM | 158 | Vocational status | **FSS**, SDMT, EDSS, NHPT, HADS-D, HADS-A | β = -0.06 | NR | NR |
| Reese (2011) | Multiple linear regression | 144 | Total costs^‖^ | **MFIS**, EQ-VAS, BDI | β = 0.47 | NR | <0.001 |
|  |  |  | Indirect costs** | EDSS, **MFIS**, EQ-VAS | β = 0.17 | NR | >0.05 |
|  |  |  | Drug costs^††^ | Age, EDSS, **MFIS** | β = 0.16 | NR | <0.05 |
| Strober (2018) | Forward stepwise logistic regression | 40 | Employment status | **MFIS-physical**, Humor, Behavioral Disengagement | OR = 0.83 | NR | 0.012 |
| Van der Hiele (2014) | Logistic regression | 44 | Employment status | SF-36, DIP, **FIS** | OR = 1.00 | NR | 0.96 |
| Van der Hiele (2018)* | Logistic regression | 161 | Being in employment | Interferon-beta, glatiramer acetate, fingolimod or any DMD use, age, gender, disease duration, number of relapses in the past year, presence of comorbidity, EDSS, SDMT, **MFIS** | OR = 0.91^§^ | NR | <0.001 |
|  |  |  | Presenteeism |  | OR = 1.08 ^§^ | NR | 0.001 |
|  |  |  | Work ability |  | OR = 0.93^§^ | NR | 0.001 |
|  |  |  | Work difficulties |  | OR = 1.75^§^ | NR | 0.001 |
| van Gorp (2018) | Spearman’s correlation | 163 | Capability set (CSWQ) | NA; correlation with **MFIS** | ρ = -0.34 | NR | ≤0.01 |
|  |  |  | Overall capability item (CSWQ) |  | ρ = -0.44 | NR | ≤0.01 |
| van Gorp (2019) | Logistic regression | 110 | Deterioration of employment status | Complex attention, executive functioning, self-reported cognitive functioning, HADS-D, **MFIS,** EDSS | OR = 0.56 | 0.27, 1.18 | 0.13 |
| Wickström (2014) | Pearson’s correlation | 58 | Change of sickness benefit | NA; correlation with change in **fatigue (FSMC)** | r = 0.13 | NR | 0.35 |

*Conference abstract

† Unclear if fatigue was continuous or categorical

‡"Fatigue is strongly associated with high sick leave”; statistical significance unknown.

§Value was calculated. Only B coefficient reported in study

¶ Only reported R^2^

‖ Includes indirect costs, drugs, hospital, rehabilitation, outpatient treatment, special equipment, ancillary therapy, copayments,etc.

**Indirect costs were related to premature retirement, disablement, unemployment, or sick leave of a multiple sclerosis patient.

††Obtained from the official German drug price list, including a deduction according to the German social security code.

Abbreviations: BDI = Beck Depression Inventory; CES-D = Center for Epidemiologic Studies Depression Scale; CI = confidence interval; CSWQ = Capability Set for Work Questionnaire; DIP = Disability and Impact Profile; DMD = disease-modifying drugs; EDSS = Expanded Disability Status Scale; EQ-VAS = EuroQoL-visual analogue scale; FIS = Fatigue Impact Scale; FSMC = Fatigue Scale for Motor and Cognitive functions; FSS = Fatigue Severity Scale; HADS = Hospital Anxiety Depression Scale; MFIS = modified FIS; MS = multiple sclerosis; MSIS = Multiple Sclerosis Impact Scale; NA = not applicable; Neuro-QoL = Quality of Life in Neurological Disorders; NFI-MS = Neurological Fatigue Index – multiple sclerosis; NHPT = Nine Hole Peg Test; NR = not reported; OR = odds ratio; PDQ = Perceived Deficit Questionnaire; PHQ-9 = Patient Health Questionnaire-9 – major depressive disorder module; PR = prevalence ratio; PROMIS = Patient-Reported Outcomes Measurement Information System; PSS = Perceived Stress Scale; QoL = quality of life; SEM = Structural Equation Model; SDMT = Symbol Digit Modalities Test; SF-36 = 36-item Short Form Health Survey; STAI-Trait = State-Trait Anxiety Inventory; UK-NDS = UK-Neurological Disability Scale; WPAI:GH = Work Productivity and Activity Impairment Questionnaire
